# Chemical proteomics approaches for identifying the cellular targets of natural products

**DOI:** 10.1039/c6np00001k

**Published:** 2016-04-21

**Authors:** M. H. Wright, S. A. Sieber

**Affiliations:** a Department of Chemistry , Technische Universität München , Lichtenbergstraße 4 , 85748 , Garching , Germany . Email: megan.wright@tum.de

## Abstract

Covering: 2010 up to 2016

Deconvoluting the mode of action of natural products and drugs remains one of the biggest challenges in chemistry and biology today. Chemical proteomics is a growing area of chemical biology that seeks to design small molecule probes to understand protein function. In the context of natural products, chemical proteomics can be used to identify the protein binding partners or targets of small molecules in live cells. Here, we highlight recent examples of chemical probes based on natural products and their application for target identification. The review focuses on probes that can be covalently linked to their target proteins (either *via* intrinsic chemical reactivity or *via* the introduction of photocrosslinkers), and can be applied “*in situ*” – in living systems rather than cell lysates. We also focus here on strategies that employ a click reaction, the copper-catalysed azide–alkyne cycloaddition reaction (CuAAC), to allow minimal functionalisation of natural product scaffolds with an alkyne or azide tag. We also discuss ‘competitive mode’ approaches that screen for natural products that compete with a well-characterised chemical probe for binding to a particular set of protein targets. Fuelled by advances in mass spectrometry instrumentation and bioinformatics, many modern strategies are now embracing quantitative proteomics to help define the true interacting partners of probes, and we highlight the opportunities this rapidly evolving technology provides in chemical proteomics. Finally, some of the limitations and challenges of chemical proteomics approaches are discussed.

## Abstract 2

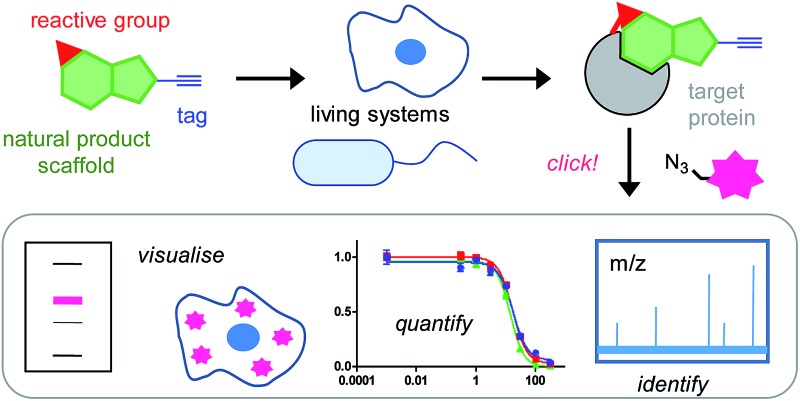
This review focuses on chemical probes to identify the protein binding partners of natural products in living systems.

## 1 Introduction

Natural products (NPs) have always been the inspiration for the development of drugs and a source of tools to elucidate cellular mechanisms. Drug discovery typically takes one of two approaches: a target-based approach, where a compound is developed to target a particular enzyme, and phenotypic screening, where libraries of small molecules are screened against live cells with a phenotypic read-out. A target-based approach has the advantages that mode of action is, in principle, known right from the outset, enzyme assays are typically reliable and easy to perform, and compound design is often aided by structural biology. Phenotypic screening, on the other hand, already takes issues such as cell uptake and efflux into account, and non-specific cytotoxicity is more easily assessed. For the phenotypic approach in particular, it is very challenging to discover and validate the mode of action of a compound. An added complexity is that compounds may have different modes of action in different organisms or contexts, or may have pleiotropic effects.

Chemical proteomics is a rapidly evolving area of chemical biology that draws on synthetic chemistry to generate probes that inform on small molecule mode of action and protein function. In the context of understanding the mode of action of NPs and other small molecules, chemical proteomics primarily provides information on the proteins a compound interacts with. Chemical proteomic strategies for NP target identification were reviewed in 2010 (ref. 1 and 2) and several reviews since have touched on the different approaches for target identification, of natural products, drugs and other molecules of interest.^3–5^ Here we aim to survey and analyse studies from the last five years on chemical proteomics technologies for mode of action, focusing on the following aspects: natural products, ‘discovery’ chemical proteomics (*i.e.* where the mode of action and binding partner of the NP is unknown at the outset), *in situ* approaches using covalent or photoprobes, two-step labelling protocols that exploit bioorthogonal ligation chemistry, and finally the advantage that can be taken of recent advances in quantitative mass spectrometry-based proteomics methods. We define ‘*in situ*’ chemical proteomics as labelling with a ‘free’ probe in living cells, to distinguish it from classical affinity chromatography (where lysates are incubated with resin functionalised with the NP of interest and binding partners retained, whilst non-binders are washed away), or incubation of cell lysates with probes. Whilst affinity chromatography has been widely and, in some cases, very successfully applied for target identification, it suffers from several disadvantages: weak but true binders are often missed (false negatives), the *in vitro* setting is more artificial than applying a probe in live cells (and therefore potentially prone to false positives), and the ‘modification’ to the probe is large and may perturb protein binding. Incubation of a free probe with lysates avoids some of these issues but not the artificial setting; approaches that apply a probe *in situ* have the significant advantage of being more biologically relevant. However, it is generally accepted that in order to be used *in situ*, the probe should be functionalised to covalently link with binding partners. This can be accomplished by incorporating either an intrinsically chemically reactive group or a photoreactive group within the probe structure.

We will first cover some general points on probe design, validation and chemical proteomics workflows. The second part of the review covers recent case studies and we conclude with a discussion on the advantages, limitations and challenges of these approaches.

### 1.1 Two-step activity and affinity-based protein profiling

#### 1.1.1 Chemical proteomics and bioorthogonal ligation chemistry

**Fig. 1:**
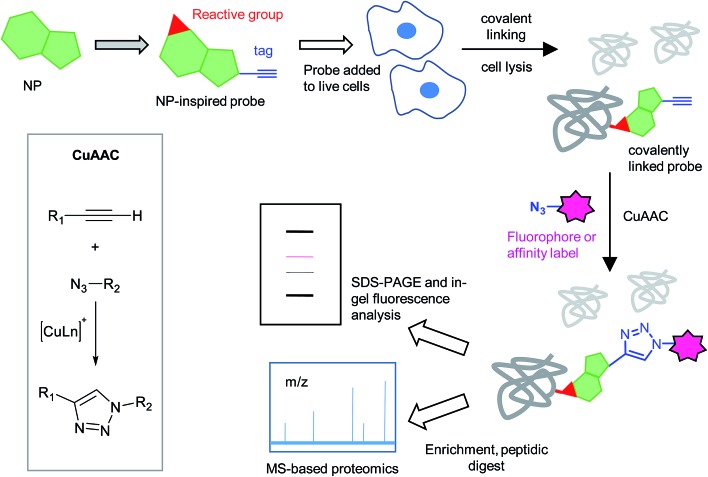
Compound-centric chemical proteomics approach for identifying the targets of natural products (NPs). The probe is designed based on the structure of the NP, is added to live cells and binds to its target protein. It reacts covalently (*via* an electrophilic trap or a photocrosslinking group) with the target protein. Cells are lysed and samples subject to copper-catalysed azide–alkyne cycloaddition (CuAAC) to attach a fluorophore or affinity label for downstream analysis.

*In situ* chemical proteomics approaches take a small molecule of interest and create a probe by functionalising it with (1) a tag that will allow downstream analysis and, if necessary, (2) a handle to stabilise the interaction between the molecule and its protein binding partner (Fig. 1). This second handle could be a photoreactive or intrinsically chemically reactive moiety. In some cases it is possible to directly attach a fluorophore or biotin affinity group in place of the tag; however, a two-step tag-then-capture approach is becoming increasingly popular. Two-step methods take advantage of bioorthogonal ligations, highly chemoselective reactions that take place under physiological conditions between two partners – probe-bound protein and fluorophore for example – in high yields and with high specificity.^6^ These reactions enable the tag incorporated into the molecule of interest to be very small: for example, a terminal alkyne, azide or cyclopropene. This approach minimises the alteration to the structure of the small molecule and therefore reduces the chances that such functionalisation will perturb binding to the target protein, as well as preserving as much as possible physicochemical properties so that cellular uptake is relatively unaffected and the probe can be applied in the context of a live cell. The most widely used bioorthogonal ligation is the copper-catalysed azide–alkyne cycloaddition reaction (CuAAC) between an alkyne and azide.^7,8^ These two functional groups are relatively easy to introduce synthetically, small, inert in biological contexts and the reaction is robust. The main disadvantage of CuAAC is that it is limited, largely, to use in lysates and fixed cells because the copper catalyst is toxic. However, for most identification strategies such toxicity is not a problem: the probe is applied in live cells (‘*in situ*’), the cells lysed and CuAAC carried out in lysate. CuAAC also denatures and, sometimes, causes protein aggregation and precipitation, which may make it incompatible with some downstream applications, although some efforts have been made to optimise reagents to reduce this effect.^9^ Two-step protocols also allow the type of label introduced to be easily varied; for example, a biotin or FLAG group can be introduced for affinity enrichment of tagged proteins and subsequent identification by standard proteomic workflows, or a fluorophore can be attached for visualisation in SDS-PAGE gels or for imaging in cells (Fig. 1). Alkynes have emerged as preferred tags due to their small size and the empirical observation that having the azide on the labelling partner during CuAAC sometimes results in lower background.^10–12^ It is worth noting that removing excess probe can sometimes be important for efficiency of CuAAC and can be accomplished by washing cells or precipitating lysates. More importantly, unreacted CuAAC labelling reagent must usually be removed before affinity enrichment analysis, since excess reagent can bind to the beads and prevent efficient pull-down of labelled proteins. This introduces an extra clean-up step, usually protein precipitation, and any additional steps in a complex workflow can introduce variability. Therefore, in some cases, direct labelling may have advantages: for example, in labelling of secreted proteins, where cell permeability of probes is less of a concern. Due to its wide applicability compared to other bioorthogonal ligations, we will focus here on CuAAC approaches for NP target identification.

#### 1.1.2 Using intrinsic chemical reactivity

**Fig. 2:**
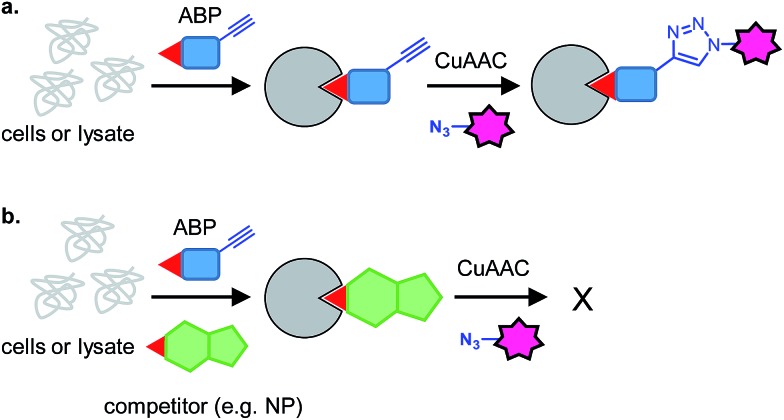
Principle of competitive ABPP. (a) An activity-based probe (ABP) reacts with target proteins and can subsequently be detected by CuAAC. (b) In the presence of a competitor, which may react with the protein or may be non-covalent, the ABP cannot bind and a reduced signal is detected.

Use of CuAAC chemistry and *in situ* labelling usually requires a second handle in the molecule to stabilise the interaction with the target protein. Pioneered by the Cravatt and Bogyo groups, activity-based protein profiling (ABPP) is now a well-established strategy that uses probes containing electrophilic groups that react covalently with enzyme active site nucleophiles.^13,14^ The reaction is ‘activity-based’ because only those enzyme molecules that are in an active conformation with an exposed active site can be labelled with the probe. Some NPs are electrophilic, exerting their biological effect *via* this type of mechanism, and are therefore readily amenable to an ABPP approach: the NP is transformed into a probe itself (Fig. 1). Alternatively, if the NP is thought to address a particular enzyme class, it can be screened in competitive mode against promiscuous ABPP probes that label that class (Fig. 2): the probe labels a defined set of proteins and if the NP addresses one or more of these by binding to the same site as the probe, a reduction in labelling of those proteins is detected. The probe may be directly functionalised with a detection tag or a two-step bioorthogonal ligation may be used. In this review we refer to the approach of modifying a NP to generate a probe as ‘compound centric’ and the approach of screening against other probes as ‘competition-based’. It is worth mentioning that not all reactive probes are strictly speaking ‘activity-based’; as illustrated later, many probes appear to not only react with active site nucleophiles but also with other residues on enzymes, and other proteins. Whether this reactivity is biologically relevant is very case- and, probably, context-dependent.

**Fig. 3:**
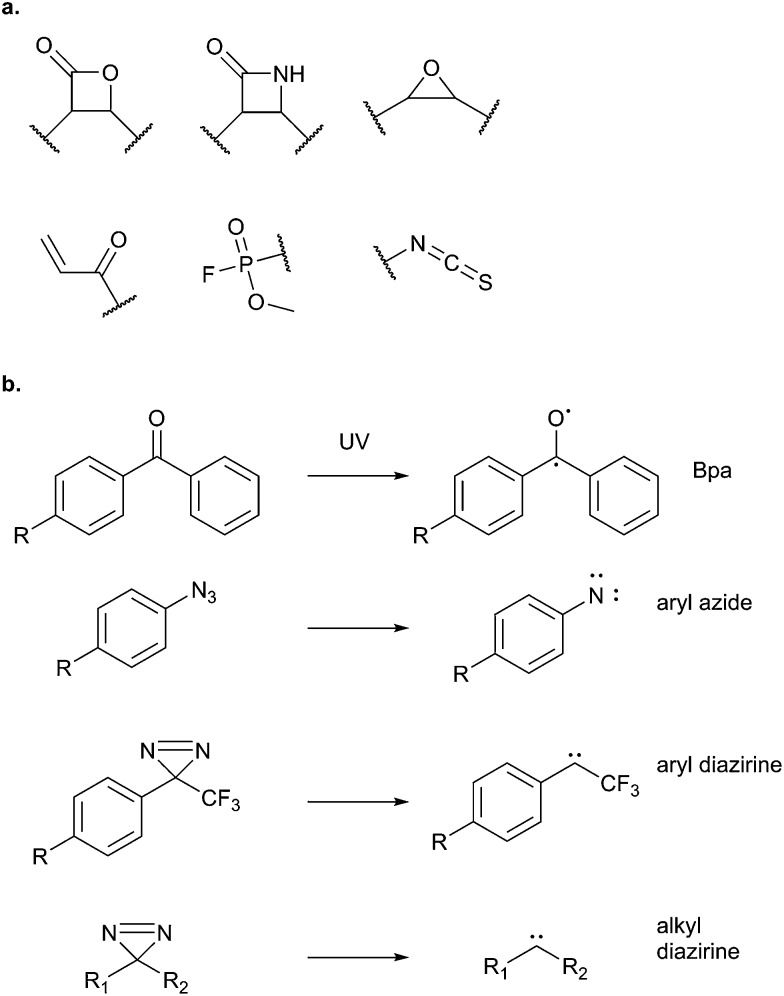
(a) Common electrophilic traps that react with nucleophilic residues in proteins. (b) Commonly used photoreactive groups and their reaction under UV light.

Potential electrophilic traps include Michael acceptors, ring strain scaffolds (β-lactones, β-lactams, aziridines, epoxides), α-halo-carbonyls, isothiocyanates and many more (Fig. 3). Some, such as β-lactones, are common motifs in natural products, whereas others, fluorophosphates for example, are not found in nature but have been discovered or designed by chemists. Tuning of electrophiles is an active area of research in chemical biology and will undoubtedly yield new reactive motifs in the future to expand the available toolbox.^15^

An ABPP approach is clearly most applicable to NPs that are intrinsically electrophilic. However, many NPs bind their targets non-covalently. An intriguing strategy, but one that has yet to be widely explored in the field, is that of ‘weaponising’ NPs with reactive traps. Porco, Cravatt and co-workers recently reported the synthesis of a β-lactone-functionalised derivative of the NP rocaglate, which was shown by competitive ABPP to target serine hydrolases.^16^ Such functionalisation of a NP has the potential to significantly alter its biological targets; however, even if this is the case, it may lead to new and unexpected useful tools for probing cellular function.

#### 1.1.3 Photoaffinity labelling

A more intuitive and unbiased strategy for identifying the binding partners of unreactive NPs is to use photoaffinity labelling (PAL). PAL makes use of photoreactive moieties that are inert under standard synthetic chemistry and biological conditions but can be activated by UV light, generating highly reactive transient species that crosslink the probe to whatever molecule is in close proximity – including presumably the protein binding partner. Commonly used PAL groups are benzophenones, aryl azides and alkyl or aryl diazirines (Fig. 3). They all have their advantages and disadvantages (reviewed in ref. 17), and a dearth of comparative studies means that no consensus has yet emerged on how to choose one over the other; indeed the choice of photocrosslinker seems to be highly context dependent.^18^ There has been a recent resurgence of interest in alkyl diazirines, paralleled by improvements in synthetic routes to access building blocks,^19^ due to their small size, unbiased reactivity, and the relatively undamaging wavelengths required for their activation.

### 1.2 Design and validation of probes

#### 1.2.1 Probe design

Designing probes based on NPs in the absence of any information on the potential targets or origin of bioactivity is very challenging, and is compounded by the structural complexity of many NPs and the necessity of developing total syntheses to access them. Even small tags and handles such as alkynes or diazirines can perturb bioactivity and binding if installed in the ‘wrong’ position. However, there is almost always scope for modifying a NP without drastically changing its mode of action and in this context structure–activity relationship information can be very helpful. An alternative approach is to generate multiple probes with tags introduced at different positions. The synthesis of tag/handle-modified NPs is highly dependent on the NP in question and the reader is referred to a recent review on the topic.^20^ Here, we will instead illustrate aspects of probe design throughout with reference to examples.

#### 1.2.2 Intrinsic reactivity of alkynes

**Fig. 4:**
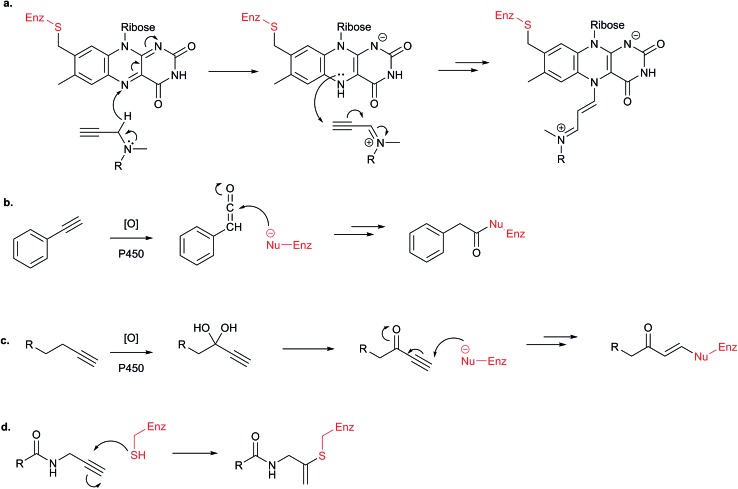
Reactive alkynes. (a) Mechanism-based inhibition of FAD-dependent enzymes by propargyl amine moiety.^21^ (b) Activation and inhibition of cytochrome p450 enzyme by aryl alkynes or (c) alkyl alkynes.^22,23^ (d) Inhibition of proteases reported by Ekkebus *et al.* ^24^

An aspect of probe design worth discussing is the potential reactivity of alkynes themselves. Terminal alkynes are found in several mechanism-based inhibitors of enzymes. One example is the inhibition of FAD-dependent enzymes by *N*-propargylamines: in the case of monoamine oxidases, the FAD cofactor, which is covalently bound to the enzyme active site *via* cysteine, oxidises the *N*-propargylamine of the bound inhibitor, and subsequently covalently attacks it (Fig. 4); this approach was used to develop activity-based probes for this class of enzymes, which are important for neurological functions due to their roles in oxidative deamination of neurotransmitters.^21^ Similarly, terminal alkynes can act as mechanism-based inhibitors of p450 enzymes: oxidation of aryl acetylenes by the enzyme generates a highly reactive ketene, which is rapidly attacked by adjacent enzyme nucleophiles. Alternatively, alkyl acetylenes can be oxidised to generate Michael acceptors. Again, such a strategy has been exploited for the design of ABPs for p450s.^22,23^ Finally, Ovaa *et al.* showed that an *N*-propargyl amide functionality attached to ubiquitin reacted selectively with the active site cysteine of deubiquitinating enzymes; furthermore, they showed that *N*-propargyl amides appended to other scaffolds also targeted the active site cysteines of other proteases highly selectively.^24^ So although alkynes are considered highly bioorthogonal in most contexts, they can be rendered reactive by enzymes or can react directly – usually aided by binding in an enzyme pocket. The extent to which alkynes react or are destroyed in living cells is currently unknown, since such reaction results in removal of the detection tag.

#### 1.2.3 Probe validation

Probes are typically validated by direct comparison with the NP of interest in bioactivity assays. Some effect on bioactivity is not necessarily damning, since the probe may be slightly more or less active than the NP but still retain the same mode of action. Full proof of probe function usually proceeds together with identification of putative binding partners.

### 1.3 Chemical proteomic workflows

With probe in hand, the first steps are typically small scale labelling experiments to optimise time, concentration and other conditions with a simple and quick read-out by SDS-PAGE gel. A typical workflow is the following: incubation of probe ‘*in situ*’, photo-irradiation if appropriate, lysis, CuAAC ligation to append a fluorophore or other detection label, and analysis by gel electrophoresis and fluorescence scanning or Western blot (to detect biotin). In-gel fluorescence is quicker and cleaner than blotting for biotin, since there are no additional signals from endogenously biotinylated proteins.^11^ Probe incubation can alternatively be carried out ‘*in vitro*’ in cell lysates. Lysis conditions are important in this case since the proteins must remain in their functional and folded states in order to specifically bind probe molecules. *In situ* approaches avoid this potential concern and probes interact with a ‘native’ system. The caveat to an *in situ* approach is that probe labelling of proteins may be reduced by uptake issues or metabolism, and cytotoxic probes can also cause problems by killing cells and reducing the amount of material recovered. The latter problem can usually be avoided by the use of short labelling times. An alternative to this compound-centric strategy is to use a competitive approach, where a NP of interest is used to outcompete promiscuous probes that, for example, bind reactive cysteines (see later). Competitive profiling is usually applied *in vitro* but has advantages in that it completely circumvents the need to create a functional probe of each compound.

The conditions for probe incubation should clearly reflect the conditions of the biological assays being used to assess the phenotype of the NP. For microorganisms the life stage or growth stage and culture medium are important, since these factors can impact uptake of compounds as well as the proteome, and hence potential probe targets. Probe concentration is also often optimised. Electrophilic and photoreactive probes usually label promiscuously at high concentrations; for example, a recent paper by Cravatt and co-workers analysed the labelling and targets of covalent kinase inhibitors converted into activity-based probes by attachment of an alkyne tag: at high concentrations of probe, labelling increased dramatically and most bands could not be out-competed with parent inhibitors – indicating non-specific reaction of probe with proteins.^25^ The authors linked this increased labelling with generic cytotoxicity. This study also illustrates how competition with parent inhibitor can be used effectively to assign proteins as true or off-targets of a probe and refine the list of hits.

#### 1.3.1 Proteomic identification of hits

To identify potential hits of probes, experiments are performed with click chemistry ligation to an affinity label – usually biotin. Biotin binds strongly to the protein streptavidin and its variants, allowing enrichment or pull-down of labelled proteins onto streptavidin-coated beads. Several groups have made use of customised CuAAC reagents that incorporate both biotin and a fluorophore to enable simultaneous enrichment and facile detection.^10,26,27^ Enriched proteins can be eluted from the beads with denaturing conditions for gel-based proteomics, or directly digested on-bead with proteolytic enzymes. In a standard gel-based proteomics workflow, enriched samples are separated by gel, bands or slices cut and digested, usually by trypsin. This approach has been widely used historically and enables simple fractionation of the sample, but suffers from contamination with environmental proteins such as keratin and problems of bias – certain proteins simply do not separate well on a gel. It is also difficult to quantify between samples. For these reasons, and enabled by the improved sensitivity of mass spectrometers, gel-free approaches are becoming increasingly common. Direct on-bead digest is simple to perform, minimises environmental contamination and loss by reducing sample handling, and is less biased than gel-based methods. In both cases, the resulting mix of peptides can be analysed by standard shotgun LC-MS/MS workflows: peptides are separated by reverse phase chromatography, and injected on-line into a tandem mass spectrometer for measurement of ions, fragmentation and measurement of fragments. Identification of proteins is carried out by software packages that compare the acquired spectra with spectra obtained from *in silico* digest of proteins in a database (*e.g.* the total proteome of the organism of interest). A vehicle control (often DMSO) is processed in parallel to provide a benchmark list of proteins that bind non-specifically to the beads. Hits are therefore identified as those proteins enriched over background.

#### 1.3.2 Quantitative chemoproteomics

Related to the shift to gel-free methods, recent chemical proteomic workflows often incorporate quantification: for example, to compare vehicle control and probe-incubated sample, competition with NP, different but related probes, biological conditions, replicates, and so on. Popular quantification approaches include SILAC (stable isotope labelling by amino acids in culture), iTRAQ (isobaric tag for relative and absolute quantitation), TMT (tandem mass tag), dimethyl labelling (DiMe), and label-free methods, such as spectral counting and MS^1^ intensity-based methods.

**Fig. 5:**
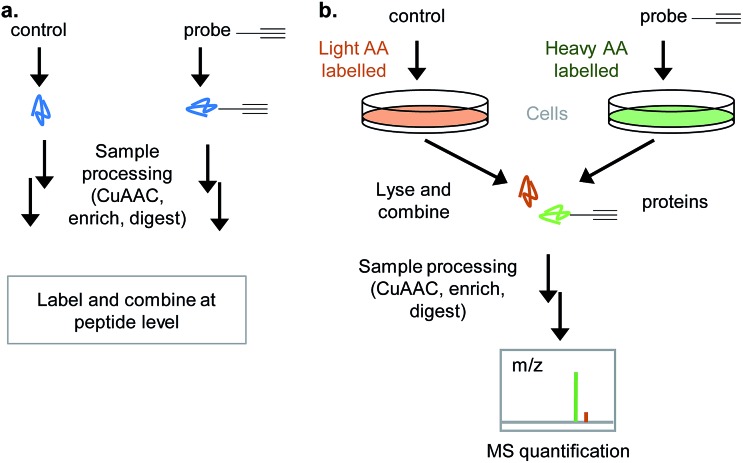
Quantitative approaches for chemical proteomics experiments. (a) In many label-based approaches (iTRAQ, DiMe…), samples are processed separately, peptides are isotope labelled and then combined before mass spectrometry. (b) SILAC (stable isotope labelling of amino acids in culture): cells are cultured with medium containing heavy or light isotope labelled amino acids; cells are treated differently (*e.g.* probe or vehicle control/untreated), lysed and samples combined for all downstream analysis.

Labelling approaches rely on the incorporation of isotopes into peptides that are then mixed and analysed together in the same LC-MS/MS run – peptides originating from different samples can then be distinguished in the mass spectrometer. Label-free approaches compare samples that are analysed in different runs and have greatly improved in recent years with the increasing sensitivity and resolution of LC-MS/MS as well as advances in computational algorithms.^28^ DiMe, iTRAQ and TMT all involve chemical labelling of peptides^29^ and so have the drawback that samples are only combined towards the end of the chemical proteomics workflow (Fig. 5a), therefore not controlling for operator error or reproducibility of the protocol to that point. Label-free suffers from the same drawback. SILAC uses cultured cells where all proteins incorporate heavy, light or medium amino acids and is therefore often a popular choice where it is applicable, since it allows samples (*e.g.* DMSO vehicle control and probe) to be combined directly after lysis (Fig. 5b). One disadvantage of labelling methods like SILAC is that only a limited number of samples can be combined; however, a ‘spike-in’ approach can be used to overcome this: in this approach a heavy standard sample is spiked into any number of samples which are run separately and then the standard used later in analysis for quantitative comparison.^30^ Considerations in the choice of quantitative approach include: the complexity and robustness of the workflow (early mixing is an advantage), the number of samples to be analysed and their origin (*e.g.* vehicle control *vs.* probe *vs.* probe + competitor in a well-studied cell culture line is easily done *via* SILAC, but for comparing tens of clinical samples from diverse origins label-free may be more appropriate), cost (some labelling methods use expensive reagents), access to MS measurement time (label-free requires each sample to be measured separately whereas in labelled approaches samples are combined), and the MS instrument set up (some methods quantify at the parent ion MS^1^ level, others at the MS^2^ fragment ion level; reproducible chromatography and instrument performance are particularly important for label-free). For a full discussion of these issues the reader is referred to several recent reviews.^29,31^

In chemical proteomics, a quantitative approach is a significant improvement on simply determining the presence or absence of a protein in a particular sample and also allows for greater flexibility in the types of experiments that can be performed. Some probes are highly specific, whereas others will label many proteins, either due to promiscuity of the NP itself or to limitations in the methodology (non-specific photoreactivity or intrinsic reactivity). We and others have found that quantifying the proteins enriched in the presence and absence of a competitor such as the parent NP, for example, can provide a lot of information on the putative hits of a probe.

#### 1.3.3 Cleavable linkers in chemical proteomics

**Fig. 6:**
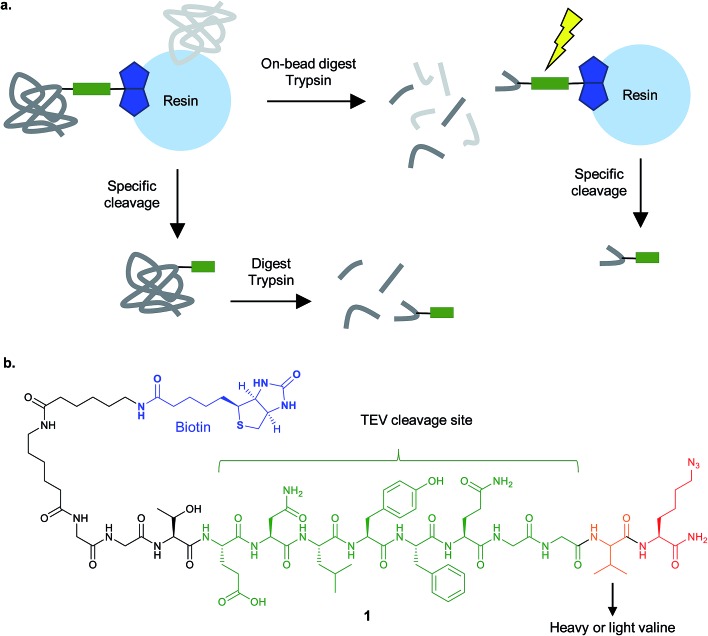
Cleavable linkers for chemical proteomics. (a) Proteins enriched on resin *e.g.* by biotin–streptavidin, can be specifically released and then analysed by MS (bottom), or digested on bead and then the modified peptide selectively released (top). (b) The isotopically-encoded protease (TEV)-cleavable linker **1** for CuAAC and enrichment reported by Cravatt and co-workers.^40^

In the majority of chemical proteomics experiments, binding of a probe to a protein is inferred indirectly from the presence of the protein (in fact its constituent peptides). This is because the probe-modified peptide is likely left attached to the beads after digest (in gel-free approaches) and/or the peptides are not separated well by HPLC or do not ‘fly’ well in the mass spectrometer due to the bulky modification. However, direct identification of the modification site adds confidence in the assignment of the protein as a hit and provides additional information about the putative binding site of the NP. Several reagents for CuAAC have been reported that incorporate a chemically, light, pH, enzyme or otherwise cleavable linker, enabling selective release of the probe-modified peptide from the beads, either with the total shotgun mix or in a separate step. Such approaches also have the potential to reduce contamination with non-specific background bead binders, since proteins linked *via* the CuAAC reagent can be selectively eluted. An early approach by the Cravatt lab termed TOP (Tandem Orthogonal Proteolysis)-ABPP^32,33^ uses click reagent **1** (Fig. 6) incorporating a TEV (tobacco etch virus) protease cleavable sequence that allows release of modified peptides from the beads orthogonal to trypsin (the enzyme typically used for the digest of proteins for MS). This reagent has been used successfully for identifying cysteines modified by simple electrophiles, such as an alkynylated iodoacetamide derivative.^34^ Diazobenzene-based reagents cleaved by treatment with Na_2_S_2_O_4_ have been used to identify peptides incorporating bioorthogonally tagged amino acids^35^ and trypsin protease cleavable linkers were recently reported for the detection of modification sites after incorporation of alkyne-tagged fatty acids or AMP analogues into proteins.^36,37^ Other reagents such as vicinal diol linkers cleavable with sodium periodate have been shown to reduce non-specific bead background^38^ but have not yet been used for site identification. In general, few cleavable reagents have been applied to directly identify the site of modification of NPs, probably because the structural complexity, large size or lability of some NP probes is still a limiting factor for their direct detection. We have also found no report of direct detection of photolinked probe in a chemical proteomic context. This is perhaps unsurprising since photoprobes can in principle react at almost any position, with coincident losses or rearrangements, yielding a very complex problem for the search algorithms. Elution efficiency of the intact proteins for gel electrophoresis is also reported to be poor in some specific cases.^39^

#### 1.3.4 Alternative enrichment/detection strategies

**Fig. 7:**
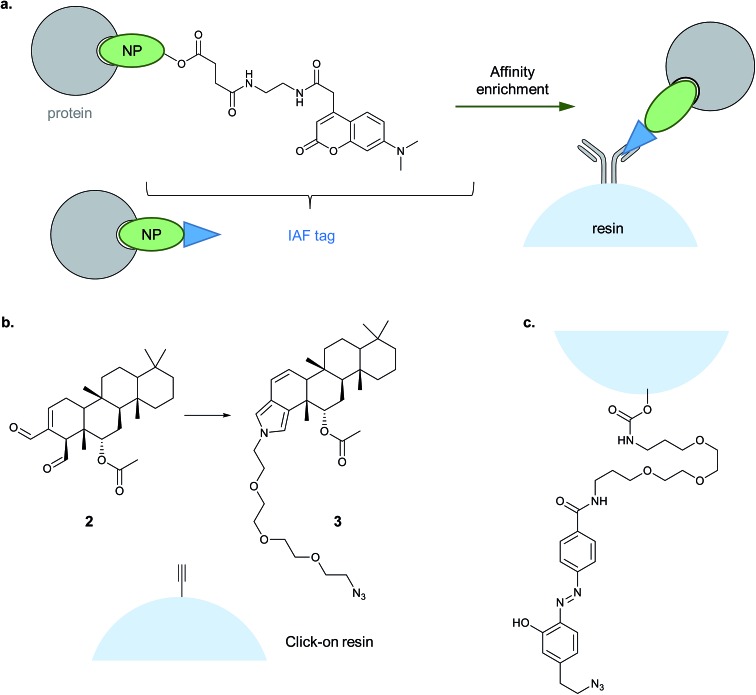
Alternative enrichment methods to biotin–streptavidin. (a) IAF (immunoaffinity fluorescent tag) reported by La Clair *et al.* for antibody-based enrichment of probe-bound proteins. (b) Click-on-resin approach reported for the NP (natural product) scalaradial: scalaradial **2** is functionalised to generate probe **3**, which can be attached in turn by CuAAC to an alkyne-functionalised resin.^45^ (c) On-resin cleavable linker reported by Sibbersen *et al.* ^46^

Biotin–streptavidin is the most widely used enrichment system, due to the strength of the interaction and the ready commercial availability of reagents. However, this system does suffer from background binding of endogenously biotinylated proteins that can hinder detection of low abundance hits. An alternative immunoaffinity method developed by the La Clair group uses a coumarin label (immunoaffinity fluorescent label, IAF), which has a dual role as both a dye for cell imaging and an affinity label (Fig. 7).^41,42^ The specific antibody raised against the coumarin epitope was shown to enable much cleaner immunoprecipitation than biotin–streptavidin in a BSA (bovine serum albumin protein) test system.^41^ In the same study the authors systematically examined CuAAC two-step labelling for both biotin and IAF, with the azide and alkyne partners on either the protein or the labelling reagent. They observed better results with the protein-alkyne and IAF-azide combination, in line with other studies.^10–12^ Interestingly, they noted that CuAAC likely requires a covalent linkage between probe and target protein for effective enrichment, consistent with the denaturing nature of the reaction: in applying their method to the NP glycyrrhetic acid, if the coumarin label was ready-attached to the probe, enrichment was possible, but two-step CuAAC did not result in any enrichment. The IAF method has been applied to identify the targets of the NPs seriniquinone^43^ and spirohexenolide A,^44^ amongst others.

Another approach that avoids biotin used a resin pre-coupled to an alkyne for simultaneous click and enrichment of an azide-tagged probe and its binding partners.^45^ The NP scalaradial **2** was functionalised with an azide, generating probe 3, added to lysates or live cells, and then lysates incubated with the alkyne-resin and click reagents (Fig. 7). The authors also directly compared the new method with a purely *in vitro* method where scalaradial was directly attached to a resin and incubated with lysates. Western blot confirmed that the previously reported protein targets PRX1 and 14-3-3ε were enriched by both methods, and gel-based proteomics was used to identify possible hits. Since scalaradial presumably binds its protein targets non-covalently, this study suggests it may be possible to optimise on-bead click to work without introducing a photocrosslinker or reactive moiety. However, controls performed in the study showed that even in the absence of the reducing agent required for the click reaction, some PRX1 and 14-3-3ε were detectable on the beads. These proteins are abundant and it remains to be seen whether such an approach will work for low abundance protein binders of a probe.

Sibbersen *et al.* recently reported a method combining click-on-resin with a chemically cleavable diazobenzene linker to facilitate release of enriched proteins.^46^ The amine- and azide-functionalised diazo-unit was prepared by coupling to NHS-activated sepharose resin (Fig. 7). The authors then applied their approach in several test systems and in comparison with either a standard biotin reagent or a diazo-biotin. They labelled cells with a promiscuous electrophilic probe, 2-oxohex-5-ynal, performed CuAAC and enriched proteins from lysates. All three methods performed comparably, giving a similar pattern of enriched proteins by Coomassie staining, with the difference that the methods employing biotin also resulted in contamination with significant amounts of monomeric streptavidin. It will be interesting to see whether this type of reagent proves useful for chemical proteomic identification of protein binders of a probe, and whether it proves possible to isolate non-covalent probe–protein adducts.

Several methods for on-resin capture and release have also been reported for peptides^47,48^ and, most recently, a silane-based fluoride cleavable linker was used to capture by strain-promoted azide–alkyne cycloaddition (SPAAC) and then release a small molecule and the enzyme chymotrypsin.^49^ Interestingly, the released enzyme still retained ∼67% of its activity, consistent with the milder nature of SPAAC compared to CuAAC.^49^

Biotin–streptavidin continues to be the most widely used method for enrichment due to the strength of the interaction and the ready availability of tools. Disadvantages include difficulties in distinguishing non-specific streptavidin background binders from true hits and challenges of identifying modified peptides. Numerous cleavable linkers have been reported to tackle the second problem, but further advances in sample preparation and bioinformatics workflows will likely be needed to expand their use. New technologies for enrichment are always a welcome addition to the chemical proteomic arsenal.

### 1.4 Target validation and mode of action

Once potential hits have been identified, the challenging tasks of validating that they are true hits and linking binding to mode of action begin. The first problem is, relatively, simple and is typically approached in several ways: if an antibody is available or can be produced, labelling can be validated by Western blot to show that the protein of interest is enriched after CuAAC and affinity pull-down, or by immunoprecipitation and on-bead CuAAC to install a label for detection. Alternatively, the protein of interest can be obtained recombinantly and labelling shown on the purified protein or the protein in the context of the overexpression system or spiked into a lysate. Labelling of an isolated protein should be viewed with caution, however, as some promiscuous probes label almost any protein in such an artificial environment. To demonstrate that probe binding is dependent on a native, properly folded protein, a ‘heat’ control in which proteins are first heated with SDS to unfold them is sometimes used. Access to the purified protein also provides the opportunity to detect modification of the protein by the probe *via* intact protein MS or to digest and identify the binding site by LC-MS/MS in a simpler system than the total enriched proteome. Some probe–protein interactions can be studied by biophysical methods, such as ITC (isothermal titration calorimetry) and structural biology can also provide supportive evidence, in the form for example of a crystal structure of probe-bound protein. In the case of electrophilic probes, if the site of modification can be identified, the amino acid residue involved, *e.g.* Cys, Ser, can be mutated and the mutant protein analysed to demonstrate that it no longer binds the protein of interest.

Demonstrating a direct link between NP binding to a protein and mode of action of the NP can be very challenging. We will illustrate the types of experiments used to tackle this problem *via* specific examples. However, in general it is necessary to apply many different biological and chemical tools. For example, in the context of the chemical proteomics experiment, access to a structurally similar but biologically inactive NP and/or probe is one approach that can help distinguish biologically irrelevant but nonetheless genuine binders from those protein targets that mediate the bioactivity. Small libraries of probes, competition experiments, comparison of labelling in biological systems where the phenotype of response differs, chemical or genetic knockout or knockdown, are all used to demonstrate a link between binding and bioactivity.

## 2 Fishing for targets: probe-centric approaches

The following section discusses specific examples where CuAAC together with *in situ* chemical proteomics has been used to identify the binding partners of NPs.

### 2.1 Early *in situ* approaches to identify the targets of electrophilic natural products

#### 2.1.1 Antibacterials

Prior to 2008, several ABPP-type approaches for identifying the targets of electrophilic NPs were reported, but studies typically used a fluorophore or biotin directly appended to the NP scaffold for detection. In 2008 we took inspiration from the fact that several classes of important antibiotics contain electrophilic β-lactone and β-lactam moieties to develop “minimal”, alkyne-tagged ABPs from these structures.^50–52^ Based on the β-lactam core, probes derived from penicillin, aztreonam, cephalosporin (**4**) (Table 1), as well as more simplified structures, were synthesised.^50^ Labelling with these probes was profiled by in-gel fluorescence after ‘*in situ*’ incubation with live bacterial cells and ‘*in vitro*’ incubation in cell lysates. Gel-based proteomics was used to identify several penicillin-binding proteins as targets, as well as a β-lactamase enzyme; this enzyme class is important for antibiotic resistance in pathogenic bacteria. Recombinant overexpression and analysis of the catalytic activity of the β-lactamase confirmed that the probes indeed inhibited the enzyme. Subsequent studies used NP-inspired β-lactams to identify and compare enzymes in antibiotic-resistant and sensitive strains of *Staphylococcus aureus*, the cause of major MRSA-hospital-acquired infections.^51^ An identified MRSA-specific enzyme of unknown function was further characterised as a likely metallo-β-lactamase by enzyme assays, labelling with other tools specific for this enzyme class, and competition of labelling with structurally-unrelated protease inhibitors. Others have also applied β-lactam probes to label the nucleophilic active sites of other enzyme classes such as a specific lysosomal cysteine hydrolase,^53^ or have developed cephalosporin C-based probes for imaging.^54^ Simplified probes incorporating the 3-membered ring heterocycles oxirane, thiirane and aziridine – other electrophilic motifs found in NPs – were also shown to label proteins *in situ* in pathogenic bacteria strains,^55^ further expanding the toolbox of probes for functionally characterising enzymes in diverse systems.

**Table 1:** NP-inspired probes applied for target protein identification studies using gel-based proteomics. Blue: modification to introduce bioorthogonal alkyne tag

Natural product and structure of inspired probe	Application, target(s)	Ref.
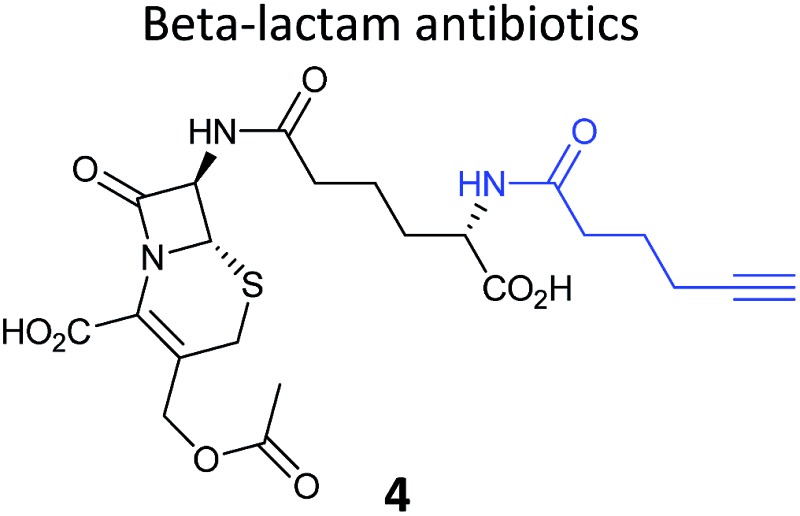	Pathogenic bacteria	50
	Penicillin binding proteins and β-lactamase identified as targets	

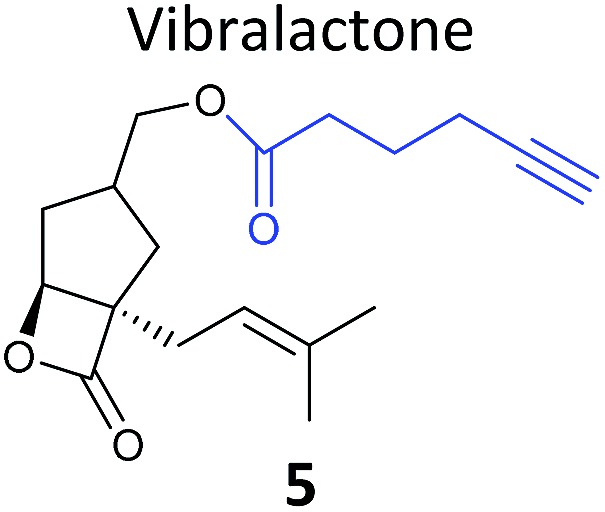	*Listeria monocytogenes*	56
	Multiple targets, including ClpP	

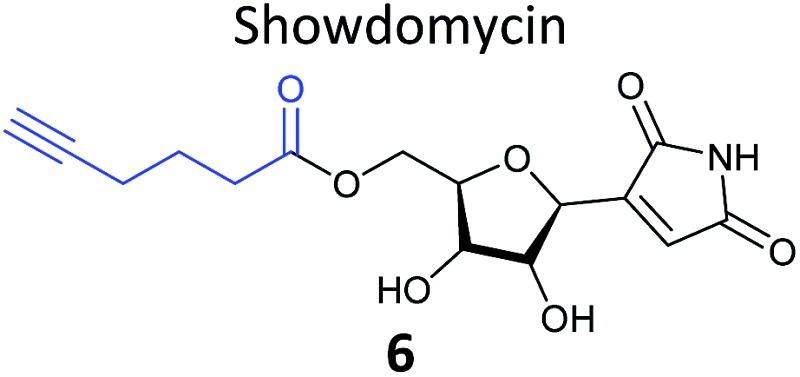	Pathogenic bacteria	58
	Targets: oxidoreductases and transferases, including the enzyme MurA1	

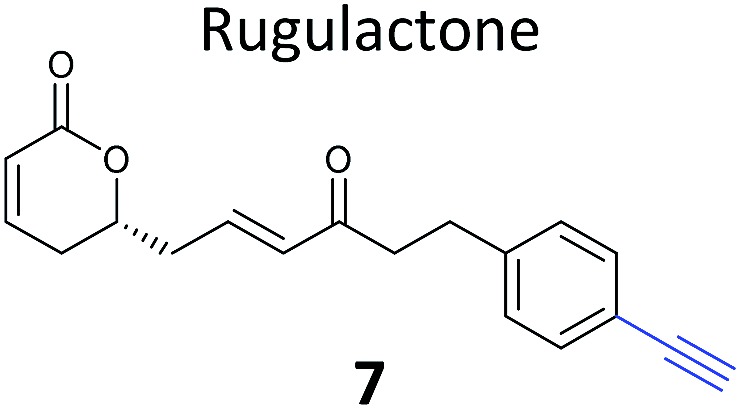	Pathogenic bacteria	59
	Targets: multiple, including kinase ThiD	

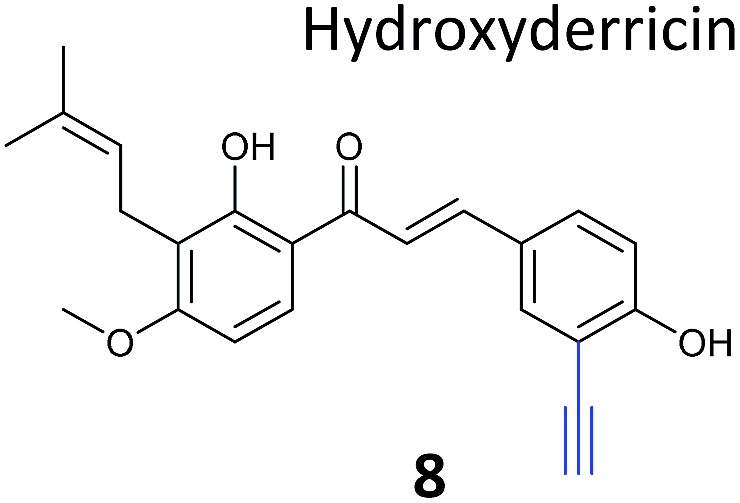	*S. aureus*	60
	Targets: multiple, including seryl-tRNA synthetase	

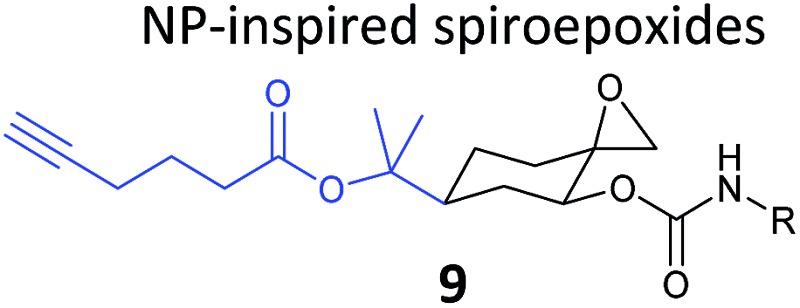	Human breast cancer cells	62
	Target: phosphoglycerate mutase 1 (PGAM1)	

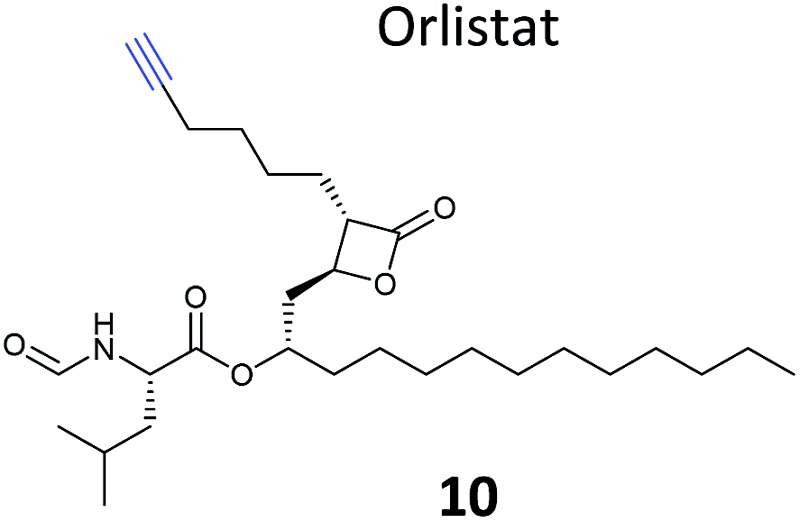	HepG2 liver cancer cells	63
	Targets: multiple, including FAS (fatty acid synthase) and GAPDH	

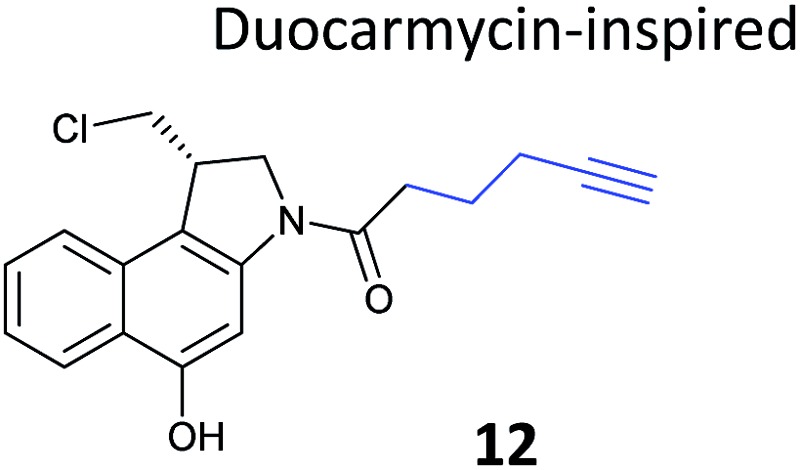	A549 cancer cells	67
	Target: aldehyde dehydrogenase ALDH1A1	

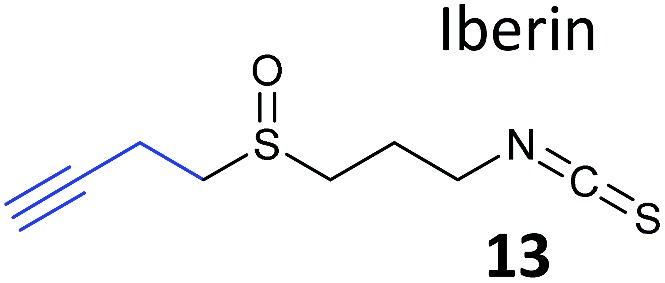	HEK293 cells expressing TLRs	70
	No proteomics – immunoaffinity methods identified binding to TLRs	

One of the interesting targets of β-lactones identified in the original study by Böttcher and Sieber was the caseinolytic peptidase ClpP, whose activity is also linked to virulence.^52^ Some organisms, such as the human pathogen *Listeria monocytogenes*, have two isoforms of ClpP, although most β-lactones only label one. Zeiler *et al.* found that an alkynylated probe **5** based on the NP vibralactone labelled both isoforms, providing a useful tool for biochemical and structural characterisation of the ClpP system in *Listeria*.^56,57^ Our group has applied a similar ABPP approach to identify the targets of the antibacterial NPs showdomycin, rugulactone and hydroxyderricin. Showdomycin is a nucleoside antibiotic with an electrophilic maleimide moiety, a motif that commonly reacts with thiols. The probe **6** was synthesised by esterification of the free alcohol on the ribose (Table 1) and applied *in situ* in different pathogenic bacteria.^58^ Proteins identified as targets by gel-based proteomics were oxidoreductases and transferases, including the enzyme MurA1, which catalyses an essential step in cell wall biosynthesis. Another putative target, the reductase AhpC, was recombinantly expressed, incubated with showdomycin, digested with chymotrypsin, and the peptides analysed by MS to identify the binding site of the NP.

The NP rugulactone also contains two, presumably cysteine-reactive, Michael acceptor systems. Nodwell *et al.* synthesised a rugulactone probe **7** possessing an alkyne tag introduced on the phenyl ring (Table 1) and a number of analogues with the Michael system reduced to assess the role of these electrophilic moieties.^59^ This is an example of a strategy used to link the reactivity of a NP with protein binding and biological activity. Application of *in situ* labelling and gel-based proteomics led to the identification of a kinase, ThiD, involved in the biosynthesis of thiamine, as one of the prominent hits. By showing that antibacterial activity of the NP increased in thiamine-deficient medium, the authors provided evidence that the bioactivity is related, in part at least, to ThiD inhibition. Battenberg *et al.* also applied ABPP to identify the targets of the antibacterial chalcone 4-hydroxyderricin (probe **8**, Table 1), another Michael acceptor functionalised NP.^60^ Labelling in the cell lysates of pathogenic bacterium *S. aureus* and gel-based proteomic identification facilitated by a diazobenzene-based cleavable linker to release bound proteins from streptavidin resin, revealed seryl-tRNA synthetase (STS) as one of the putative hits. The enzyme, which is responsible for loading cognate tRNAs with serine, was inhibited *in vitro* by the NP, suggesting it is a functionally relevant target. However, identification of which of the five cysteines in STS is targeted by the probe proved difficult, suggesting that the compound may bind to multiple nucleophilic sites on the enzyme.

#### 2.1.2 Cytotoxic and anti-cancer NPs

The ABPP approach has also been applied to identify the targets of reactive NPs in human cells. Inspired by NPs such as fumagillin and Lumanicin C, Cravatt and co-workers synthesised a library of alkynylated probes containing a reactive spiroepoxide moiety (*e.g.* **9**, Table 1) and screened them for cytotoxicity.^61^ The compound with the highest cytotoxicity in a human breast cancer cell line was further shown to label an additional band compared to biologically inactive probes in *in situ* profiling experiments. This band was identified as a glycolytic enzyme phosphoglycerate mutase 1 (PGAM1) by gel-based proteomics. Interestingly, PGAM1 was not labelled *in vitro* by the probe, highlighting the value of applying probes in a native *in situ* setting. The TOP-cleavable linker approach described above was used here on partially purified PGAM1 after labelling of the enzyme in an overexpression system, resulting in the identification of the region of modification by the spiroepoxide probe. A follow-up study clarified the site of modification as a lysine residue in the enzyme.^62^

β-Lactone probes have also been applied in human cells. The β-lactone-containing anti-obesity drug orlistat is a derivative of the NP lipstatin, an irreversible inhibitor of pancreatic lipase. To investigate the putative targets of orlistat in cancer cells, Yao and colleagues synthesised orlistat-like probes incorporating an alkyne handle at different positions (*e.g.* **10**, Table 1).^63^ The anti-proliferative activity and cellular effects of probes were analysed in HepG2 liver cancer cells, confirming their bioactivity was comparable to that of orlistat, and then the probes were applied in labelling experiments. Labelling patterns *in vitro* were comparable to *in situ*, but background labelling was higher *in vitro*. Gel-based proteomics identified known target FAS (fatty acid synthase) as well as additional putative targets of the drug. Western blotting with antibodies after pull-down confirmed the enrichment of several hits, and some of these were overexpressed and shown to be labelled by the orlistat probe. A Cys–Ala mutant of identified putative target GAPDH, an abundant housekeeping protein, was not labelled by the probe, indicating probe labelling of this particular cysteine residue. GAPDH and additional proposed targets of orlistat have yet to be linked to mode of action of this drug, but at the very least orlistat-based probes have revealed that the compound covalently reacts with a number of cellular targets. In 2014, the Wenk and Yao groups further applied orlistat probes to investigate the bactericidal activity of the drug.^64^ Gel-based proteomics after probe labelling of *Mycobacterium* cell lysates revealed lipid esterases as targets.

**Fig. 8:**
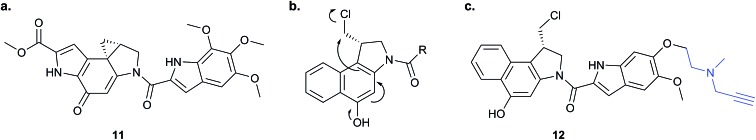
(a) Cytotoxic NP duocarmycin **11**. (b) Seco-drug version of duocarmycin and activation mechanism. (c) Duocarmycin-inspired probe **12** reported by Wirth *et al.* ^67^

Chemical proteomics can reveal unexpected modes of action of NPs. Duocarmycins (*e.g.* **11**, Fig. 8), a class of anti-tumour NP from *Streptomyces*, are known to act by alkylation of DNA *via* an electrophilic cyclopropane and derivatives use a seco-drug structure where the cyclopropane is formed *in situ* from an appended chloromethyl (Fig. 8).^65^ However, removal of the indole DNA-binding unit, gave a compound that was still highly cytotoxic, raising the possibility of alternative non-DNA related targets.^66^ Wirth *et al.* synthesised a duocarmycin-inspired alkynylated probe **12** which retained cytotoxicity and labelled one prominent protein after *in situ* incubation with A549 cancer cells.^67^ In yet another example of such an effect, the probe barely labelled this band in cell lysates. Gel-based proteomics identified the protein as aldehyde dehydrogenase ALDH1A1. A follow-up study applied additional probes lacking the DNA-binding motif as well as those based on a bifunctional scaffold; these probes also labelled ALDH1A1.^68^ Further crystallographic, computational and biochemical studies of ALDH1A1 clarified the mechanism of inhibition and the binding site of duocarmycin-inspired compounds.^69^

Food-derived natural products with potential anti-inflammatory properties are also interesting bioactive compounds. A recent study looked at the isothiocyanate iberin, which was identified from cabbage and onion extracts as an inhibitor of inflammation-mediating toll-like receptors (TLRs).^70^ As part of this wider study, alkynylated probes (**13**, Table 1) were synthesised and applied *in situ*. Target engagement was confirmed using Western blot for TLRs after enrichment of tagged proteins, although additional targets of the iberin probes were not identified by proteomics.

### 2.2 Going gel-free and quantitative

A survey of the examples given above makes it clear that although some reactive NPs are highly selective for a few targets, many probes label multiple proteins. Distinguishing between true hits related to bioactivity, hits that are irrelevant for bioactivity but nonetheless genuinely labelled, probe-specific off-targets, and background proteins from CuAAC or enrichment, is not trivial. As discussed previously, the increasing sensitivity and resolution of mass spectrometers, coupled with the improvement in algorithms for analysing complex datasets, has brought quantitative proteomics to the fore in chemical biology. Gel-free approaches enable unbiased identification of hits and quantification allows for far more sophisticated experimental set-ups, including measuring binding *in situ* by quantifying increasing concentrations of probe, comparing across different tissue or cell types, or quantifying response to inhibitors or competitors. The following recent examples of NP or NP-inspired probes highlight the use of gel-free and/or quantitative proteomics to study NP mode of action.

#### 2.2.1 Studies using label-free methods

Pyrethroids are widely used insecticides derived from pyrethrins, botanical NPs, and act by blocking voltage-gated sodium channels. Ismail *et al.* developed a library of pyrethroid-inspired alkyne probes in order to profile the cytochrome p450 (CYP) detoxifying enzymes that may be involved in pyrethroid metabolism and resistance.^71^ Aryl-alkynes are known mechanism-based inhibitors of CYPs (as described above) and probes incorporated an alkyne warhead as well as a second alkyne for click chemistry (*e.g.* **14**, Table 2). Incubation of mouse liver microsomes with probes and gel-based identification with semi-quantification by a gel-free method revealed the profile of CYPs labelled. This was followed up with gel-free analyses, which gave a similar list of CYP targets, although their rank differed compared to the gel-based data. The semi-quantitative method used, emPAI (Exponentially Modified Protein Abundance Index),^72^ is based on coverage of the protein sequence and was used here to indicate the relative abundance of different enzymes. Importantly, the gel-free approach enabled the identification of additional drug-metabolising enzymes labelled by pyrethroid probes.

**Table 2:** NP-inspired probes applied for target protein identification studies using gel-free and quantitative proteomics. Blue: modification to introduce bioorthogonal alkyne tag

Natural product-inspired probe	Application, methods, target	Ref.
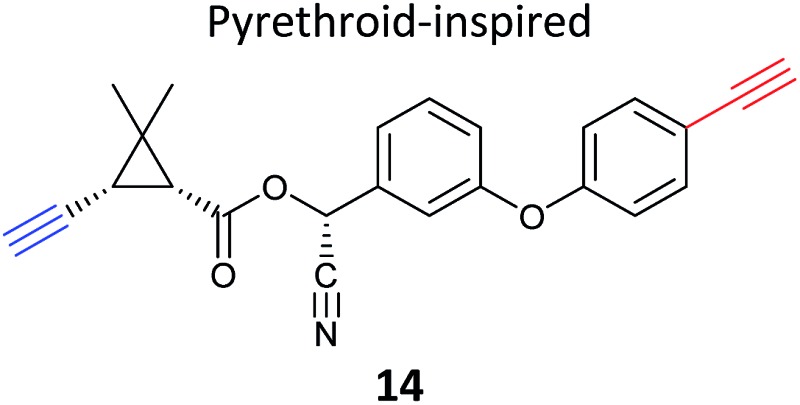	Mouse liver microsomes	71
	EmPAI quantitative proteomics	
	Targets: cytochrome p450 enzymes	

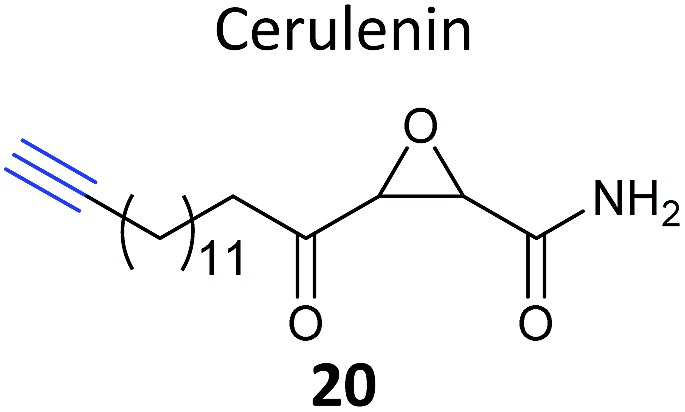	Melanoma cells and Hek293 cells overexpressing PAT enzymes. Quantification by spectral counting	77
	Multiple targets, including PATs, likely palmitoylated proteins	

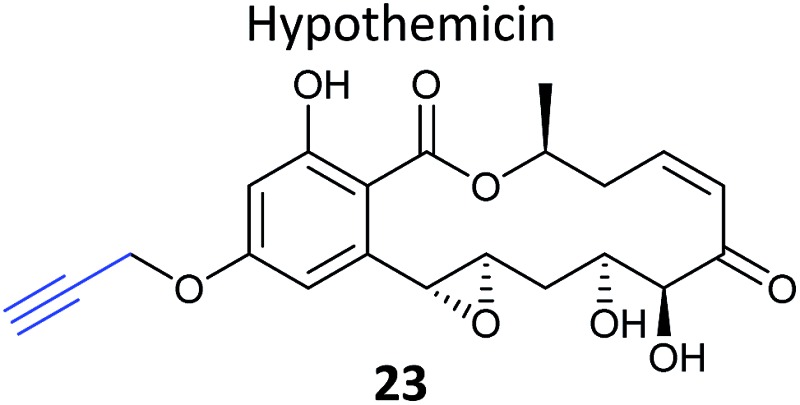	*Trypanosoma brucei*	80
	Gel-based and gel-free iTRAQ	
	Targets: kinases, including TbGSK3short, TbCLK1 & 2	

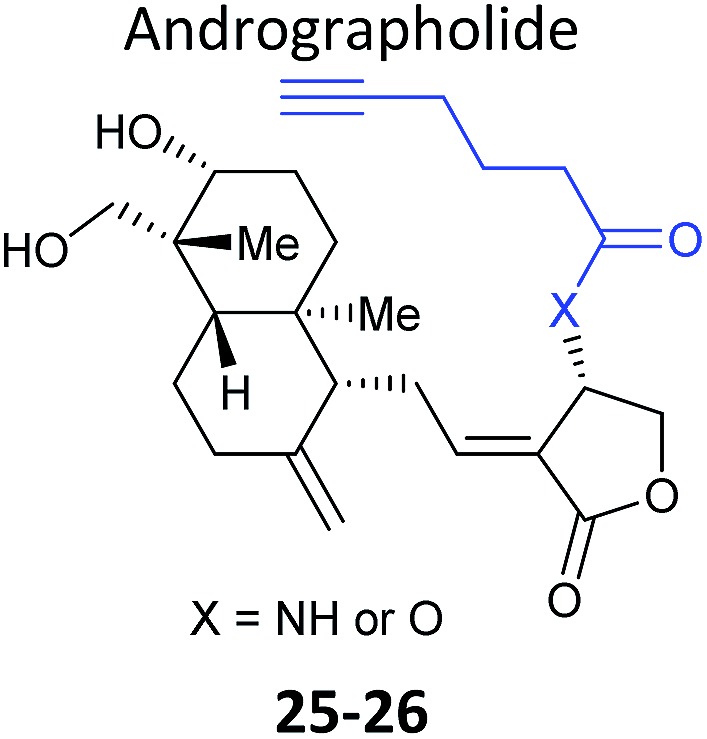	Human cancer cell lines	81
	iTRAQ proteomics	
	Targets: multiple	

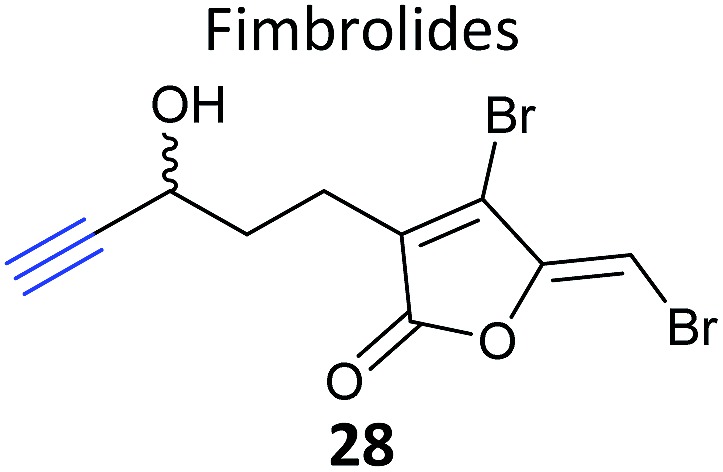	*Vibrio* species	85
	Dimethyl labelling proteomics	
	Targets: LuxS and LuxE, others	

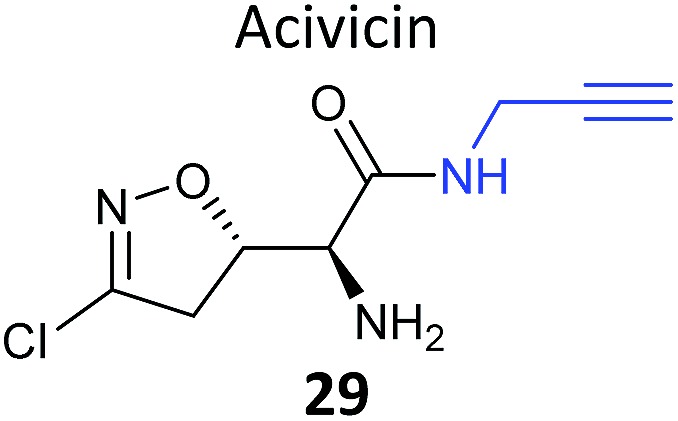	Liver cancer cell lines and mouse liver tissue	87
	SILAC proteomics	
	Targets: multiple, including aldehyde dehydrogenases (esp. ALDH4A1) and CES1 (metabolite target)	

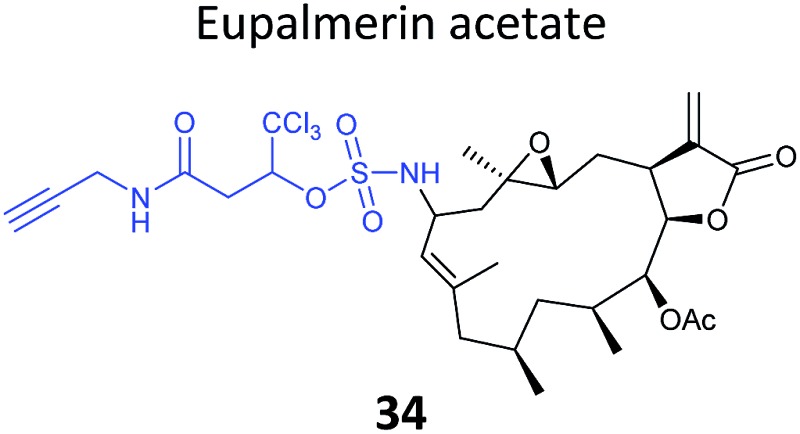	HL-60 leukaemia cells	89
	SILAC proteomics	
	Targets: DERL1, CYB5B, TBXAS1	

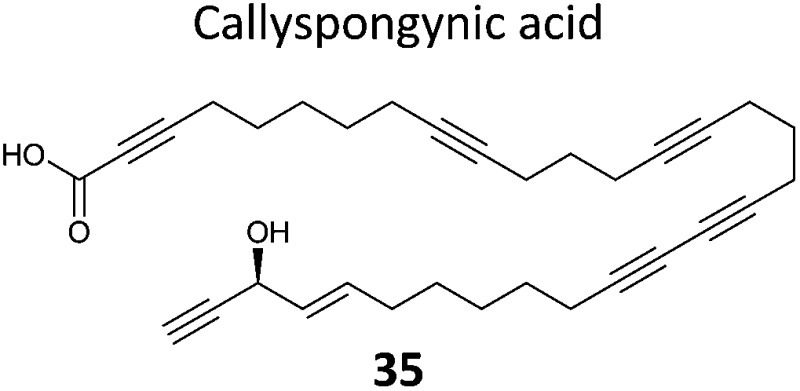	HeLa and HEK293 cancer cells	90
	SILAC proteomics	
	Targets: multiple membrane-associated proteins and proteins involved in lipid biosynthesis/metabolism	

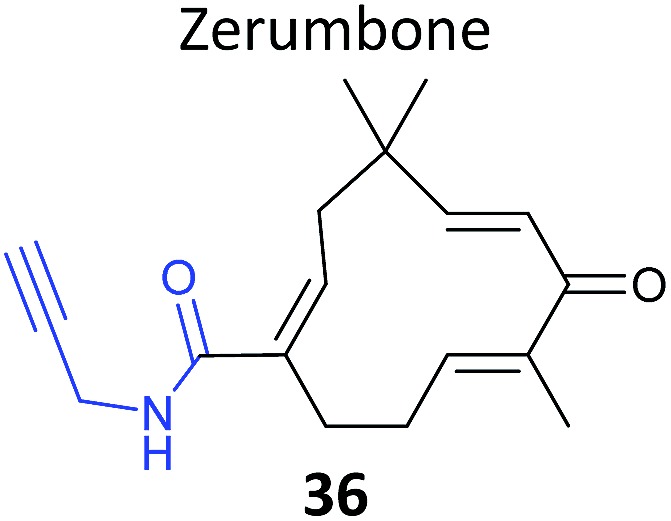	HeLa cancer cells	91
	Spike-in SILAC proteomics	
	Targets: multiple	

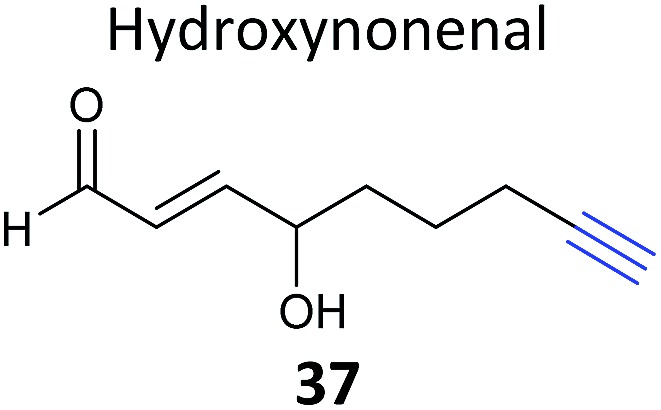	RKO colon cancer cells	92
	Peptide-centric approach; quantification based on isotopically-tagged, UV-cleavable CuAAC reagents	
	Targets: multiple-reactive cysteines	

**Fig. 9:**
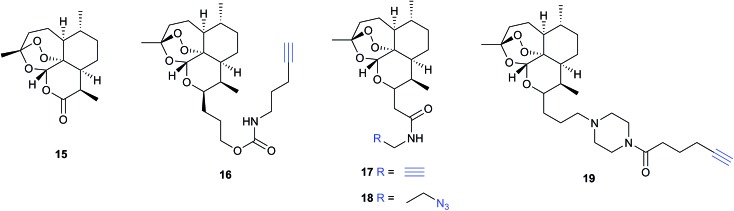
Antimalarial natural product artemisinin (ART, **15**) and reported probes **16–19**.^73–75^

Several recent studies have used chemical proteomics to profile the modification of proteins with the important antimalarial drug artemisinin (ART). ART is a plant-derived natural product with an unusual endoperoxide bridge (**15**, Fig. 9); this feature is thought to be activated by iron species in the red blood cells where the malaria parasite resides, generating reactive radicals that can attack cellular structures or biomolecules. However, the identities of modified proteins and the mode of action of ART are not fully understood. Wang *et al.* ^73^ and Ismail *et al.* ^74^ independently reported several alkyne- or azide-tagged artemisinin probes (**16–18**, Fig. 9); these were incubated with parasite cultures, proteins captured by CuAAC, enriched and identified, with semi-quantification *via* emPAI scoring in both cases. Wang *et al.* identified 124 enriched proteins and showed that several of the enzymes were inhibited by activated artemisinin *in vitro*; they further applied their alkynylated probe to study the mechanism of ART activation (an open question in the field) using iron chelators or inhibitors of haem production, concluding that haem is the dominant source of activation. Ismail *et al.* applied azido- and alkynyl-probes to identify around 60 enriched proteins; they also studied the effect of free iron chelators, observing some effect on labelling but also noting that this was not sufficient to explain ART modification of several proteins. Wang *et al.* also applied their probe in a human cancer cell line, since cancer cells have been proposed to be more susceptible to ART treatment than normal cells. A third recent study (Zhou *et al.*) also identified ART targets in cancer cells using an alkynylated probe **19**, similarly concluding that haem is the agent responsible for ART activation.^75^ Together these three studies shed light on the likely protein targets of ART in live cells and provide tools to probe biological questions of the mode of action of this important drug.

Another label-free approach is spectral counting; this method estimates protein abundance by counting the number of MS^2^ spectra assigned to a particular precursor ion and hence protein, although the method suffers from limited dynamic range and can only accurately quantify abundant proteins in a sample.^76^ Zheng *et al.* recently used spectral counting to quantify the chemical proteomic targets of an alkyne-tagged version of the NP cerulenin (**20**, Table 2).^77^ Cerulenin has been proposed to inhibit palmitoylacyltransferase (PAT) enzymes, which carry out the lipid modification of proteins known as palmitoylation, and fatty acid synthases by reaction of the α-keto-epoxide with active site cysteine residues. Zheng *et al.* modified the terminus of the long aliphatic chain of the NP with an alkyne, and carried out identification of targets of the cerulenin probe in melanoma cells. They compared this with cells pre-treated with cerulenin itself or another PAT inhibitor 2-bromopalmitate in competition experiments. Several proteins that responded to competition were indeed identified and further experiments validated that two of these were palmitoylated. The authors also profiled the labelling pattern in lysates from Hek293 cells transfected with different members of the DHHC family of PAT enzymes; however, mutation of the active site cysteines of DHHC 3 and 4 did not abolish labelling, suggesting that the probe labels other sites and therefore is not ‘activity-based’.

**Fig. 10:**
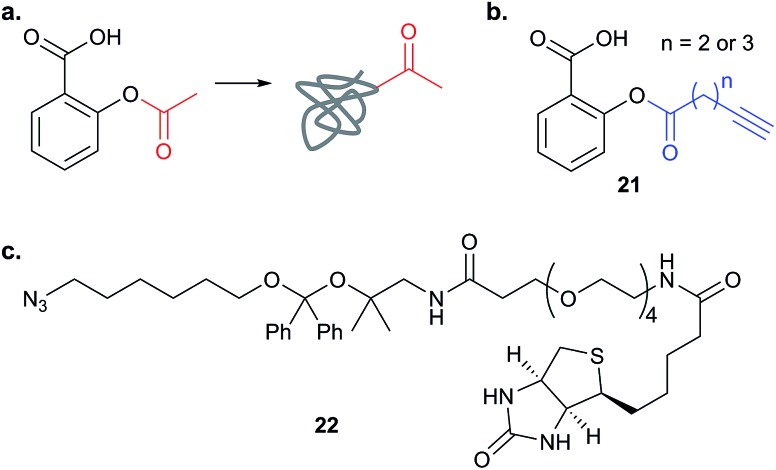
(a) The NP aspirin alkylates proteins. (b) Structure of aspirin-based probes **21** that have been reported.^78,79^ (c) Cleavable linker **22** for the detection of aspirin probe-acylated peptide sites.^79^

Spectral counting was also used by Pratt and co-workers in a study with alkynylated aspirin (**21**, Fig. 10).^78^ Well known as an anti-inflammatory drug, aspirin acts in part as an acetylating agent, covalently modifying key serine residues in cyclooxygenase enzymes, but the identities of other potential acetylation targets were unknown. HCT-15 colorectal cancer cells were labelled with aspirin probe and proteins identified by a standard chemical proteomics workflow. The authors performed three replicates and used spectral counting and other cut-off criteria to identify potential hits; acetylation was further confirmed for several histone proteins.

#### 2.2.2 Label-based approaches: quantification at the peptide level

Apart from the bias of spectral counting towards larger proteins and the difficulties of quantifying low abundance hits, it is also challenging to define a cut-off when faced with a list of proteins and counts – to differentiate specific and non-specific binders for example. This is particularly true when dealing with probes that label many protein targets, as is the case for the examples of cerulenin and aspirin discussed above. Therefore, many recent studies have used alternative quantification methods. In an independent report, Wang *et al.* also applied alkyne-modified aspirin probes to identify protein targets of aspirin acetylation in cancer cells, but used quantification by label-based method iTRAQ.^79^ In iTRAQ, isotope labelling is performed at the peptide level, in this case after peptide release by on-bead digest. Wang *et al.* also applied a cleavable linker strategy to orthogonally release modified peptides from the beads and simultaneously quantify sites of aspirin acetylation. In this case, the novel CuAAC reagent **22** contained an acid cleavable linker (Fig. 10), so that following on-bead digest and removal of most peptides, the modified peptides could be easily cleaved with 5% formic acid. For the ∼1000 proteins quantified in the study, the modified site was identified in roughly half of cases, providing high confidence for the identification of these proteins as true hits of the probe. Although lysine was the main acetylated residue, the authors also found evidence for modification of other side chains, such as serine and threonine, although the high concentration of probe used may mean that some of these are not physiologically relevant – always a concern with chemical probes. Based on their high confidence hit list, Wang *et al.* predicted the pathways affected by aspirin acetylation and showed that aspirin induces autophagy and activates lysosomal function in cells.

Natural products with validated mode of action in one organism are sometimes transferable to less well characterised systems. In 2013, the Taunton *et al.* showed that the polyketide NP hypothemycin, which is known to inhibit several human kinases, is also active in *Trypanosoma brucei*, the protozoan parasite that causes the disease Human African Trypanosomiasis (HAT).^80^ Hypothemycin contains an electrophilic Michael system which reacts with the nucleophilic thiol of select kinases. Taunton and colleagues created alkynylated probe version **23** *via* semi-synthetic modification of the NP – by simple replacement of a methyl ether with propargyl ether (Table 2). **23** had the same potency as the NP in parasite growth inhibition assays and labelled multiple bands after CuAAC. Only one clear band at 43 kDa showed dose-dependent saturation of signal intensity and could be out-competed by the parent NP, however, in an elegant example of using a gel-based read-out to assess specificity of probe labelling. Gel-based proteomics of the 43 kDa band revealed the kinase TbGSK3short as the dominant target but several other kinases were also identified. Noting that low abundance targets could be overlooked in proteomic analyses given that the probe also labelled abundant proteins non-specifically, the authors next carried out gel-free, iTRAQ-based quantitative chemical proteomics with samples incubated with their probe and increasing concentrations of the parent NP as competitor. Competition curves were then plotted with the quantified data for each protein, confirming TbGSK3short as a strong hit, but also showing that the kinase TbCLK1/2 was in fact more sensitive to modification by the NP. Follow-up experiments showed that the isoform TbCLK1, and not CLK2 or the more abundant kinase GSK3short, is likely the relevant target for parasite death. This study is therefore a nice example of how gel-free, quantitative proteomics combined with competition experiments and followed up with careful validation can deconvolute the biologically-relevant targets of a NP.

**Fig. 11:**
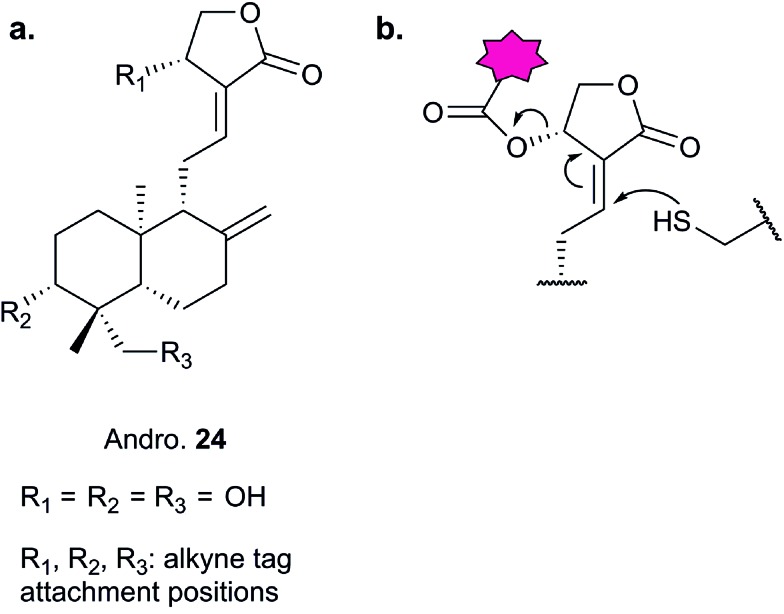
(a) Andrographolide **24** and positions used to incorporate an alkyne in various reported probes (see also Table 2).^81,82^ (b) Fluorogenic probe reported by Yao *et al.* ^82^ reacts to release a fluorophore upon attack by a nucleophilic cysteine residue.

Further lessons on the promiscuity of some reactive NPs and the challenges of identifying the interaction responsible for bioactivity are provided by the NP andrographolide (Andro **24**, Fig. 11), which has diverse anti-inflammatory and anti-cancer effects. Two independent studies have developed probes for Andro and applied them in diverse cancer cell lines.^81,82^ Andro possesses an α,β-unsaturated γ-lactone moiety and in the first study Wang *et al.* modified the pendant hydroxyl by esterification or replaced it with an amide for introduction of an alkyne (**25** and **26** Table 2).^81^ The amide probe gave far more intense labelling in cancer cell lines, likely because the ester is hydrolysed, resulting in loss of the alkyne tag; in fact elimination of the β-hydroxy group has been proposed to occur during reaction with proteins. After iTRAQ quantification of replicate experiments with the amide probe and filtering, the authors identified 75 possible hits, including one known target. Several hits were confirmed by enrichment and Western blot. Two proteins were then studied in more detail *via* overexpression and identification of cysteine residues as Andro binding sites. Finally, based on pathway analysis of identified proteins, the authors predicted that metastasis would be affected by Andro treatment; indeed, fairly low concentrations of Andro reduced migration and invasion in HCT116 cells. The second report on Andro probes took a different approach to probe design. Taking advantage of the susceptibility to elimination of any leaving groups appended to the α,β-unsaturated γ-lactone ring, the Yao group attached fluorogenic dyes at this position to provide a rapid read-out of probe reaction for imaging studies.^82^ The alkyne tag was instead attached to alcohol functionalities at the other end of the molecule (Fig. 11). Gel-based proteomics identified several hits; however, the authors also randomly selected recombinant proteins and showed that almost all were labelled by Andro *in vitro*. On this basis they concluded that Andro is a highly promiscuous compound, consistent with the study of Wang *et al.*

**Fig. 12:**
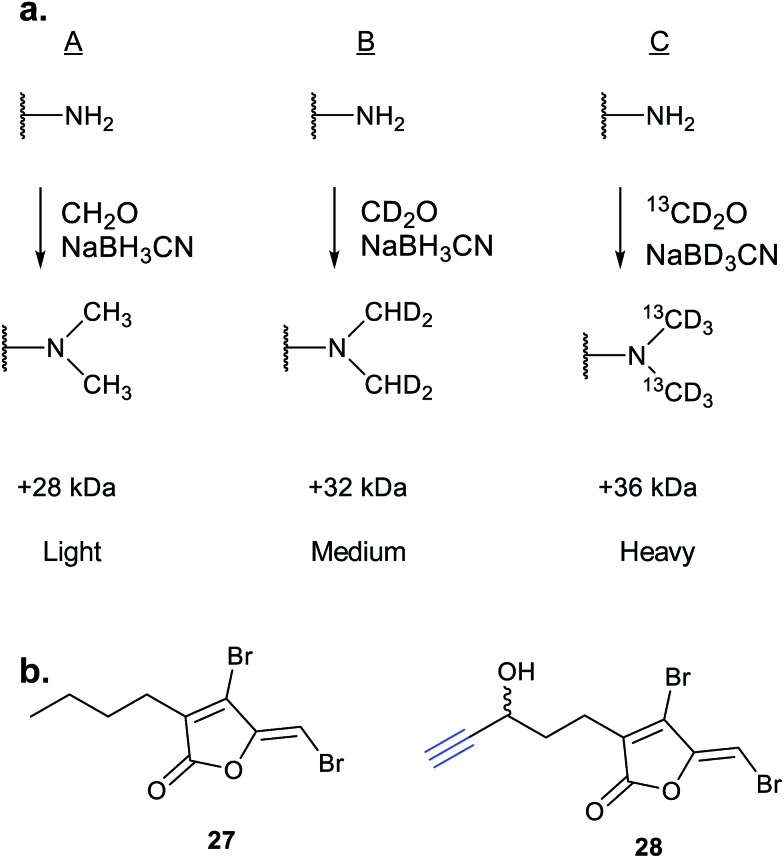
(a) Dimethyl labelling (DiMe) on peptide amines for quantitative comparison of up to 3 samples. (b) Fimbrolide **27** and corresponding probe **28**.^85^

iTRAQ uses reporter ion quantification: this means that the different reagents used to label peptides are isobaric – the same in mass – resulting in identical parent peptides which are differentiable only after fragmentation in the mass spectrometer (so-called ‘reporter ions’ are released). An alternative to iTRAQ for peptide-level mixing of samples is dimethyl labelling (DiMe), where quantification is carried out at the parent ion (MS^1^) level and which has the advantage of using inexpensive reagents.^83^ DiMe works by isotopic labelling of free amines in peptides *via* reductive amination with formaldehyde and sodium cyanoborohydride, both available with heavy isotopes incorporated such that peptides from different samples differ in mass by >4 Da (Fig. 12a). A recent example from our lab used DiMe quantification to deconvolute the targets of fimbrolide NPs. Fimbrolides such as **27** (Fig. 12b) are marine halogenated furanones that inhibit bacterial quorum sensing, intercellular communication *via* small molecules.^84^ Zhao *et al.* synthesised alkyne-tagged variants of these halogenated furanones (*e.g.* **28**, Table 2) and applied them in live *Vibrio* cells – bacteria well-studied for their ability to engage in QS-induced bioluminescence.^85^ After assessing the panel of probes for their ability to reduce bioluminescence in the bacteria, the fimbrolide and corresponding probe showing the best and most comparable activity were selected for further studies. DiMe was used to quantitatively compare proteins enriched from samples treated with (1) DMSO vehicle, (2) probe **28**, and (3) probe **28** following addition of excess NP **27** (competition). The competition experiment was used to select protein targets shared by the probe and NP, which included QS-related enzymes LuxS and LuxE. Binding of the probe to cysteine residues was further shown in these two cases *via* MS/MS analysis of digested recombinant protein. Several other targets were also identified, suggesting that fimbrolides act *via* multiple pathways in bacteria.

#### 2.2.3 Label-based approaches: SILAC

Protein quantification by SILAC, where samples can be mixed at the lysate stage, is very attractive because it does not suffer from the problem that samples are combined at the peptide level – which is the case in methods such as iTRAQ and label free described above.

**Fig. 13:**
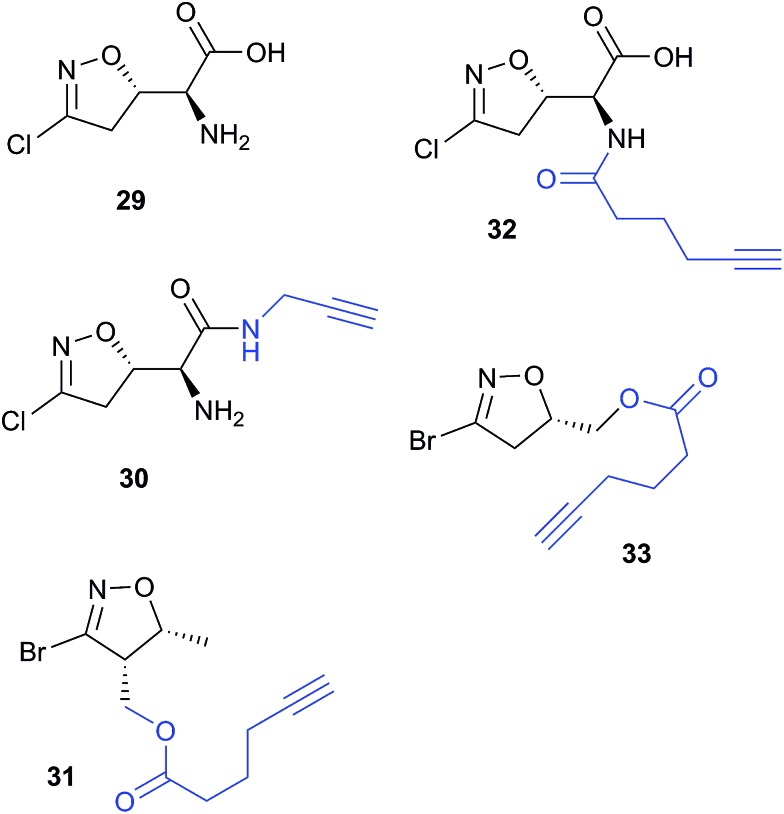
Acivicin **29** and probes **30–33**.^87^

Acivicin (**29** Fig. 13) is a NP with broad bioactivity that has been studied as a potential anti-cancer agent. Several enzymes had been identified as potentially inhibited by acivicin, but no unbiased and global analysis of potential protein targets had been carried out. The 4-chloroisoxazole moiety reacts with nucleophilic side chains in an addition–elimination reaction. An initial study by Orth *et al.* developed ACV-inspired 4-chloro- and 4-bromo-isoxazole probes and applied them in gel-based chemical proteomics analyses of potential targets in bacteria, identifying several dehydrogenases as likely targets.^86^ Kreuzer *et al.* extended this approach in a liver cancer cell line and mouse tissue.^87^ Since acivicin is a small compound with several potential sites for modification, Kreuzer *et al.* generated several different probes **30–33** to maximise the chances of identifying relevant targets. Gel-based chemoproteomics identified aldehyde dehydrogenases amongst the targets in mouse liver lysate and HepG2 human cells, and subsequent gel-based SILAC experiments, including quantification of competition with the NP, confirmed the hits in the latter. Interestingly, after prolonged incubation of cells with probe **30**, a new target, identified as carboxylesterase 1 (CES1), appeared. Recombinant CES1 itself was not labelled by **30**, however, although it was active, suggesting that a probe metabolite is the responsible agent. Follow-up *in vitro* studies and siRNA knockdown in human cells confirmed CES1 and one aldehyde dehydrogenase, ALDH4A1, as proteins important for viability.

The marine diterpene eupalmerin acetate (EuPA) shows activity against cancer cell lines and in a mouse tumour xenograft model.^88^ The mode of action of EuPA is presumed to arise from reaction of the exocyclic alkene with nucleophilic residues in proteins. Romo and colleagues explored the use of rhodium(ii)-catalysed C–H amination chemistry to remodel and functionalise the structures of complex NPs.^89^ EuPA was derivatised with an alkyne tag and the resulting probe **34** (Table 2) applied in HL-60 leukaemia cells; many bands were labelled but only a few of these could be outcompeted by the parent NP. To identify the responsive proteins, SILAC experiments were used to (a) compare heavy and light EuPA-probe-treated cells, and (b) compare cells treated with either probe alone or first treated with parent NP and then probe. Duplicate runs and a gel-free protocol were used, and hits selected on the basis of an enrichment of ∼1.0 in experiment (a), and a high ratio of probe/competition in experiment (b). The three most promising hits were further validated by overexpression and labelling in Hek293 cells.

A few natural products already contain terminal alkynes that are potentially usable as tags. Nickel *et al.* devised a total synthesis of callyspongynic acid (**35**, Table 2) to test its potential as a probe.^90^ Like other polyacetylenes, callyspongyinic acid is lipid-like and was therefore hypothesised to act on lipid signalling pathways in the cell. *In situ* labelling in human cell lines revealed a number of bands and gel-free proteomics with SILAC in triplicates was used to quantify enrichment in NP-treated cells compared to a vehicle control. The authors note the high reproducibility of SILAC ratios between replicates and that the majority of the ∼400 proteins quantified across all three replicates showed no enrichment, indicative of the very high sensitivity of modern MS instruments for detecting background binders on the enrichment beads. Gene ontology analysis of hits revealed that almost half were enzymes that could potentially bind callyspongynic acid or a metabolite for further metabolism or degradation. Non-enzyme putative targets were also identified and the authors speculate that these, mostly membrane, proteins may be enriched due to membrane perturbations of the NP.

As mentioned above, standard SILAC is limited by the available isotopically labelled amino acids to direct comparison of three samples mixed together, and cannot be used to analyse tissue samples, for example. However, use of a ‘spike-in’ standard can sometimes overcome these limitations. In spike-in SILAC a single reference sample is produced by SILAC labelling and then spiked into any number of different experimental samples.^30^ Provided the spike-in reference has appropriate coverage of the proteome in the experimental samples, the reference can then act as an internal standard, allowing comparison across all samples, which can be produced independently and from different sources. Spike-in SILAC was used by the Tate group to identify the targets of the NP zerumbone, for quantifying concentration-dependent competition of alkyne probe with the parent NP.^91^ The cyclic sesquiterpene zerumbone, which has diverse anti-inflammatory activities, possess two electrophilic Michael acceptor systems with potential protein reactivity and so a probe **36** was synthesised with an alkyne attached at a distal site (Table 2). A large batch of SILAC heavy cells were labelled with the probe, and subsequent triplicate samples in standard cell culture medium were prepared with probe and increasing concentrations of parent NP. Statistical analysis of the enrichment ratios allowed a subtle analysis of the hits to identify those where probe binding was most robustly out-competed. The profile of high confidence hits revealed proteins with diverse biological functions, underlining the likely pleiotropic mode of action of zerumbone.

#### 2.2.4 Alternative quantification approaches

All the studies discussed above performed enrichment of proteins following CuAAC and then digested these for shotgun proteomics. In select cases, orthogonal release of the modified peptide was also applied to identify the site of reaction of the probe. However, an alternative approach is to digest proteins first and then carry out CuAAC and enrichment of peptides with a cleavable reagent. Although this results in a loss of information – since the majority of the peptides from a particular protein are not present – it is one strategy for focusing on the site of modification, and enrichment of peptides is also likely to be less biased and more efficient than enrichment of proteins. A recent study by Yang *et al.* applied such a peptidic site-centric approach to identify modification of proteins by the electrophilic lipid 4-hydroxy-2-nonenal (HNE).^92^ HNE is one of a number of endogenously produced lipid-derived electrophiles which modify proteins and thereby mediate responses to oxidative stress. In 2008, an azido-tagged HNE analogue was used to identify potential targets, including several heat shock proteins, by gel-based proteomics.^93^ The follow-up study from Yang *et al.* then applied the peptide-centric approach with alkyne-tagged HNE **37** (Table 2) and novel isotopically-tagged, UV-cleavable CuAAC reagents (“Az-UV-biotin”).^92^ **37** was incubated with RKO colon cancer cells, cells lysed and proteins digested with trypsin; Az-UV-biotin reagents were then reacted with peptide mixtures, samples enriched on streptavidin beads and released by irradiation with UV light. The isotopic tagging in the Az-UV-biotin reagents facilitated identification of the peptides and quantification. As an example of how this could be applied, Yang *et al.* analysed pulse-chase type experiments in which cells were labelled with **37**, and then incubated in HNE-free medium for different periods of time. Using this approach they were able to measure the dynamics of HNE modification in live cells.

### 2.3 Photoaffinity labelling

This review has thus far focused on NPs that are intrinsically electrophilic, of which there are many examples. However, most NPs act by non-covalently binding their targets. Photoaffinity labelling (PAL) is a technology in which photoreactive groups are introduced into a probe so that a covalent link can be made between the probe and its target. PAL has been experiencing a resurgence in recent years, fuelled by the development of CuAAC chemistry allowing the introduction of minimal tags for target fishing. Benzophenone, aryl azide, aryl and alkyl diazirines are the most commonly used PAL groups (Fig. 3).^17,18^

**Fig. 14:**
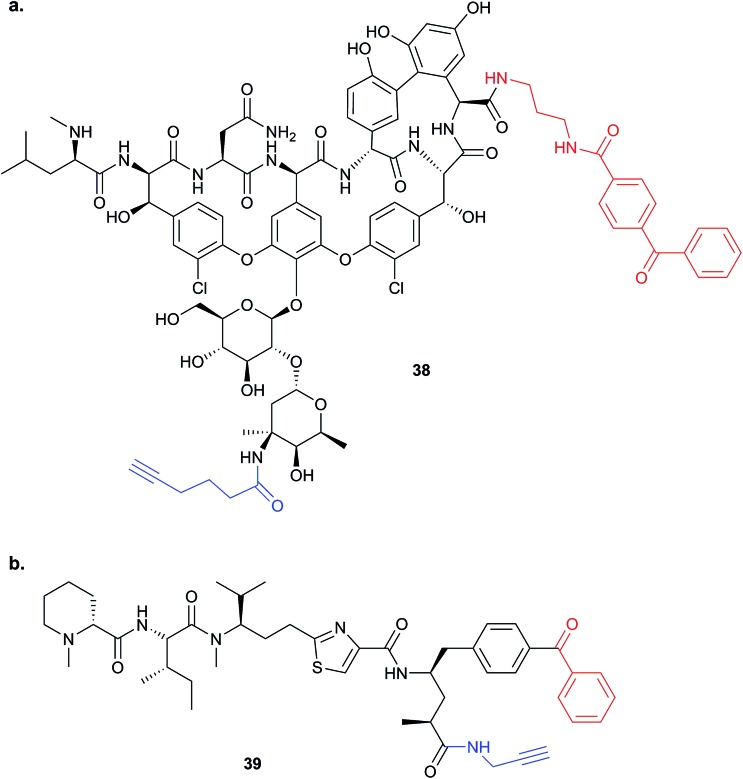
Benzophenone (Bpa)-containing photoprobes. (a) Probe **38** based on vancomycin.^94^ (b) Probe **39** based on pretubulysin.^27^

Benzophenone (Bpa) is a historically popular PAL group due to its stability towards chemical synthesis. Bpa–alkyne probes (*e.g.* **38**) of the glycopeptide antibiotic vancomycin was used to identify potential protein binders in pathogenic bacteria (Fig. 14).^94^ Eirich *et al.* synthesised several probes with the Bpa and alkyne moieties in different positions and applied them both *in vitro* and *in situ*. Two distinct protein hits were identified by gel-based proteomics in antibiotic-resistant *S. aureus* and *E. faecalis* as bifunctional autolysin (ATL) and a peptide ABC transporter respectively. Both hits were subsequently validated by recombinant expression and labelling, and the degradation of peptidoglycan by ATL shown to be inhibited by vancomycin. Eirich *et al.* reported another example of a Bpa photoclick probe **39**, based on pretubulysins, compounds derived from the NPs tubulysins (Fig. 14).^27^ Structure–activity relationships aided probe design, and the bulky Bpa was incorporated at a position known to be tolerant to modifications. Despite this careful design, there was a significant drop in activity. However, labelling was performed in HeLa cancer cells and one particularly prominent band was shown to respond to competition; this band was identified as tubulin, the known target of tubulysins. The authors further applied their probe for fluorescence imaging in cells.

**Fig. 15:**
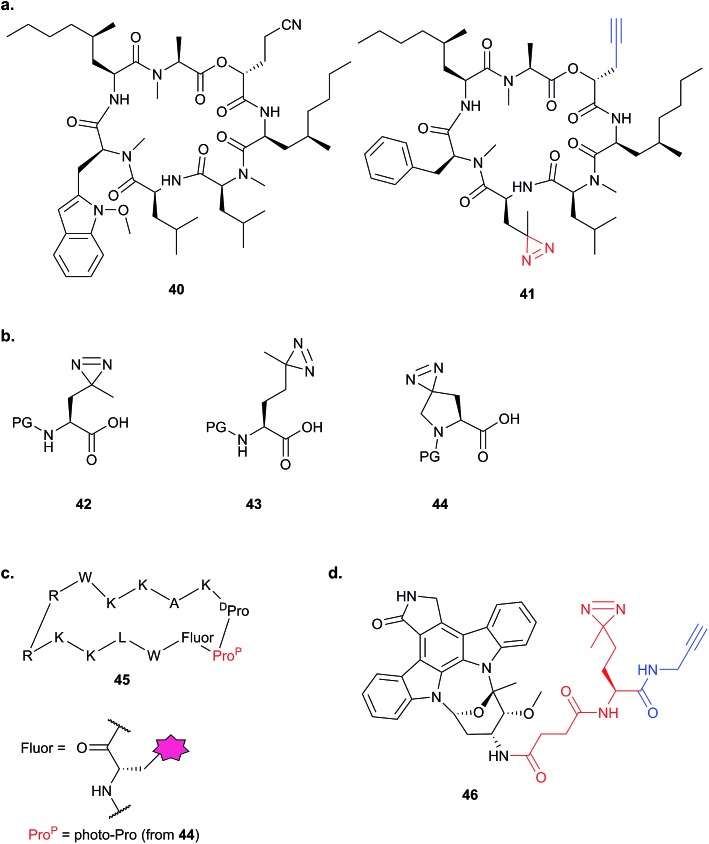
Diazirine-based photoprobes. (a) Probe **41** based on a fungal depsipeptide NP **40**.^96^ (b) Amino acid analogues photo-Leu **42**, photo-Met **43** and photo-Pro **44** incorporating diazirines. PG = protecting group. (c) Peptidomimetic probe **45**.^98^ (d) Staurosporine photoprobe **46**.^101^

The bulky size and possible propensity of Bpa to react preferentially with methionine^95^ are disadvantages. Other PAL linkers such as aryl azides are best excited by short wavelengths of light (typically 265 to 275 nm), potentially damaging proteins which absorb light in this range. The first example of incorporation of the small alkyl diazirine group into a NP was reported in 2007 by the Taunton group.^96^ In this study, a probe version of a fungal cyclodepsipeptide (HUN-7293, **40**), which was known to inhibit cotranslational translocation, was designed. The probe, **41**, contained an alkyl diazirine-tagged unnatural amino acid derivative “photo-Leu” **42** and alkyne tag (Fig. 15a and b). Crude ER microsome fractions were incubated with the probe, irradiated, denatured with SDS and ligated to a fluorescent azide derivative by CuAAC. One clear major band was observed at a molecular weight consistent with that of the hypothesised target, Sec61α, a subunit of the complex that forms the translocation channel at the ER membrane. This target was confirmed by labelling in microsomes depleted of Sec61α. The authors concluded that the use of small photo- and click-tags which presumably minimally perturb the binding of the peptide to its target, together with the photoreactive properties of the diazirine, were key factors in the success of this identification strategy.

The diazirine-functionalised amino acid derivative photo-methionine **43** has also been reported.^97^ A third unnatural diazirine-functionalised amino acid derivative, photo-proline **44**, was incorporated into a peptidomimetic with potent antibacterial activity against the Gram-negative bacterium *Pseudomonas aeruginosa* (probe **45**, Fig. 15).^98^ The parent peptidomimetic was based on the structure of the naturally occurring membranolytic host-defense peptide protegrin I. A forward genetic screen suggested an outer membrane protein as a potential target, and to confirm direct binding in live cells the authors synthesised a biotin-functionalised analogue of the photoreactive peptidomimetic and showed that it did indeed label the putative target LptD.

Alkyl diazirines have also been incorporated into non-peptidic compounds. The NP staurosporine (STS) is a potent, promiscuous kinase inhibitor, which has found use as a general probe for kinases in different technological approaches: an STS analogue has been directly attached to resin,^99^ and an aryl azide-based photocrosslinker and biotin-tagged probe synthesised for detecting binding partners in cell lysates.^100^ In order to detect kinase targets of STS in live cells, Yao and colleagues prepared an STS analogue **46** with pendant alkyl diazirine and alkyne for click chemistry (Fig. 15).^101^ Gel-based fluorescence and proteomic analysis of proteins labelled by the STS probe revealed overlapping but distinct profiles in lysates and live cells; five ‘*in situ*-specific’ targets were further validated by pull-down and Western blot. In addition to the 43 kinases identified in their approach, the authors also saw a large number of other proteins in the LC-MS/MS experiments. In this case, the authors chose to focus on kinases, although they note that kinase-related and -binding proteins could also be enriched by the probe. The Yao group expanded on this concept with the report of a series of versatile ‘minimal’ alkyl-diazirine and alkyne-functionalised building blocks that can be incorporated into scaffolds of interest.^102^ They incorporated these into kinase inhibitors, including STS, and applied a cocktail of several probes to identify kinases in different cell and tissue types. However, in addition to the 94 kinases identified *via* the probes from rat kidney tissues, hundreds of non-kinases were apparently enriched. Whether these other putative targets represent true off-targets of the inhibitors or simply probe-specific background remained unexplored. This example illustrates a very real challenge of photoaffinity labelling for discovery: how to identify true binders against a, potentially high, non-specific background. We discuss this point further in the conclusions, below.

**Fig. 16:**
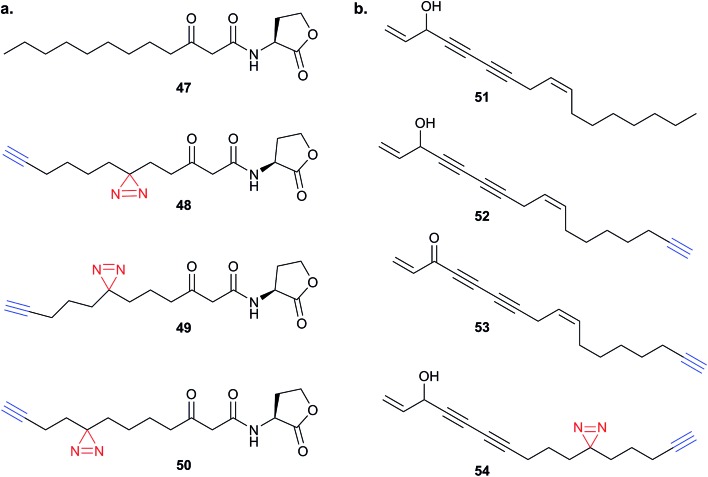
(a) Acyl homoserine lactone **47** and reported probes **48–50**.^104^ (b) Falcarinol **51** and inspired probes **52–54**.^106^

Endogenous small molecules with unknown or unclear mode of action are also the subject of studies employing PAL. Acyl-homoserine lactones (*e.g.* **47**) produced by Gram-negative bacteria are interesting compounds which, in addition to their roles in bacterial quorum sensing, also trigger responses in other organisms – such as yeast and human cells.^103^ The Meijler group reported a series of alkyne-tagged probes incorporating a diazirine in different positions (**48–50** Fig. 16a) and applied these tools to demonstrate labelling of the known bacterial target LasR.^104^ Interestingly, the phenotypic response of bacteria and human cells to the probes differed – demonstrating that even the minimal alkyl diazirine modification can have strong influences in small molecules. A further example of probes based on endogenous small molecules is the application of alkyne- and diazirine-tagged cholesterol analogues to identify sterol-binding proteins in human cell lines by Cravatt and co-workers.^105^ SILAC was used here to quantify enrichment over control, compare different probes and determine sensitivity of each protein *via* competition with native cholesterol.

Finally, our group recently applied PAL to identify the binding partners of the NP falcarinol.^106^ Falcarinol and the related NP stipudiol are extracted from vegetables and have been reported to have anti-cancer properties, but falcarinol is also known to alkylate nucleophiles and isolated proteins.^107^ Heydenreuter *et al.* synthesised the NP **51**, alkynylated probe **52** and an alkynylated version of the oxidised NP falcarinone **53** (Fig. 16b).^106^ *In situ* labelling in A549 cancer cells revealed a prominent band that was identified by gel-based proteomics as aldehyde dehydrogenase 2 (ALDH2). To further identify the possible reversible targets of the NP, the authors synthesised an alkyne probe **54** incorporating an alkyl diazirine. SILAC and gel-free MS was used to quantify the targets of the photoprobe. Several other proteins appeared in this analysis, in addition to the previously identified covalent target ALDH2; the latter was further shown to be inhibited by falcarinol *in vitro*.

## 3 Competitive activity-based protein screening

### 3.1 Screening platforms

**Fig. 17:**
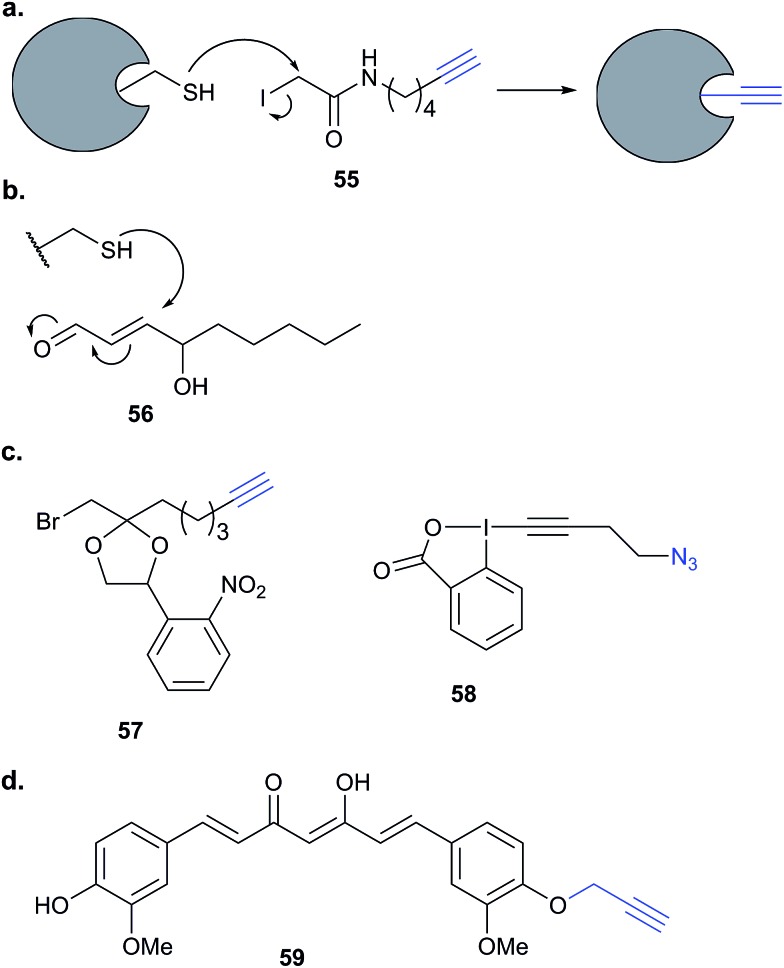
(a) Reaction of iodoacetamide alkyne probes **55** with nucleophilic cysteine residues in proteins.^34^ (b) Reaction of 4-hydroxy-2-nonenal (HNE, **56**) with cysteine. (c) Caged bromomethyl ketone electrophilic probe **57** (ref. 109) and alkynyl benziodoxolone reagent EBX, **58**, for labelling cysteines.^110^ (d) Curcumin-alkyne probe **59** for compound-centric target identification.

Historically, ABPP approaches have focused on compounds bearing electrophilic warheads that react with nucleophilic residues, often in enzyme active sites. This has led to the development of many probes that may preferentially label a specific enzyme class but are often quite promiscuous in labelling multiple proteins within that class. Many of the studies discussed above make use of competition experiments to provide evidence that a probe binds the same target as its parent compound. Combining these two concepts, small molecules of interest can be added as competitors to promiscuous probes to detect, for example, selective binders. Competitive ABPP (Fig. 2) has not been very widely applied to elucidate the mode of action of natural products. However, given the potential complexity of probe synthesis and the potential perturbations to function from introducing even minimal tags into NPs, such approaches are worth considering. Many promiscuous ABPs preferentially react with cysteine residues. An example is iodoacetamide-alkyne **55** (Fig. 17), which has been applied to profile cysteine reactive proteins in total proteomes.^34^ Probes based on phenylsulfonate esters, epoxides, Michael acceptors and α-chloroacetamides all display proteome reactivity, with a preference for functional reactive nucleophilic residues.^108^

### 3.2 Competitive mode for natural product target elucidation

Competitive ABPP can provide a different perspective and an orthogonal approach for identifying the binding partners of reactive endogenous molecules or NPs. The electrophilic lipid 4-hydroxy-2-nonenal (HNE **56**, Fig. 17) has a Michael acceptor susceptible to reaction with cellular cysteines. Several probes of HNE have been reported that incorporate an azide or alkyne tag (see previously). However, in order to rapidly screen for ‘hyper-reactive’ cysteine residues that could act as cellular redox sensors by reacting with HNE, Cravatt and co-workers applied their isoTOP-ABPP (isotopic tandem orthogonal proteolysis activity-based protein profiling) method in combination with cysteine-reactive iodoacetamide-alkyne (IAA, Fig. 17) for competitive ABPP.^40^ isoTOP-ABPP uses heavy or light versions of an azide-TEV-biotin tag for click chemistry and enrichment of labelled proteins, allowing comparative quantification of samples.^34^ Proteomes of a breast cancer cell line were treated with lipid electrophiles, including HNE, IAA added and samples analysed by gel-free MS.^40^ Of the ∼1000 cysteines quantified in the experiments, most were relatively unaffected, but a small subset responded to treatment with the lipids. To further investigate the susceptibilities of these cysteines, samples were treated with different concentrations of HNE and quantified relative to DMSO sample (here acting as the standard, by analogy with the spike-in SILAC approach). In this way, half-maximal inhibitory concentrations (IC_50_ values) could effectively be calculated. Follow-up investigations of the hit ZAK kinase revealed that HNE modification of a specific cysteine residue inhibited kinase activity. Furthermore, the previously reported alkyne-HNE **37** (Table 2)^93^ also labelled this protein, providing further evidence that it is a target of lipid electrophiles. The authors discuss the value of applying these two complementary approaches, noting that the indirect nature of IAA profiling means that the competitor may in fact not bind the same cysteine as IAA but instead an adjacent residue that is sterically shielded by probe binding.

IAA is rarely used in living cells, being instead mostly applied in proteomes due to its high toxicity and the high concentrations required to saturate labelling. The Weerapana group recently reported caged bromomethyl ketone probe **57**, that is unreactive and can accumulate in live cells with low toxicity but is then unmasked by irradiation (Fig. 17).^109^ **57** was used to profile the effects of EGF stimulation, which enhances production of reactive oxygen species. Abegg *et al.* have reported another generic cysteine-reactive probe for use in live cells based on alkynyl benziodoxolone and carrying an azide tag for click chemistry (EBX, **58**, Fig. 17).^110^ This probe proved to be more reactive than IAA and, although it was still toxic, the higher reactivity allowed lower concentrations to be used for *in situ* labelling. To demonstrate the utility of their probe, and compare it to IAA, Abegg *et al.* profiled the NP curcumin in competitive mode. Curcumin is found in the spice turmeric and has been reported to have anti-cancer and anti-inflammatory properties. The Michael acceptor of curcumin is potentially cysteine-reactive, and indeed did out-compete select bands in both IAA and EBX treated samples. SILAC chemical proteomics was used to identify those labelled cysteines selectively outcompeted by curcumin, and this was further backed up by labelling with an alkyne-tagged curcumin derivative **59** (Fig. 17). Of the 57 targets identified by competitive ABPP, 42 were also found as targets of **59**. The authors also noted that the complexity of the fragmentation patterns of alkyne-curcumin-labelled peptides meant that site identification with **59** was very challenging – whereas identifying the cysteine modification sites with the simpler IAA or EBX reagents was more straightforward. The hits identified with IAA and EBX also did not overlap completely, highlighting the value of having multiple reagents available to profile reactive cysteine residues. A further recent study independently applied the same probe **59** together with iTRAQ chemical proteomics to identify curcumin targets in colon cancer cells.^111^ The authors identified nearly 200 enriched proteins, adding further evidence that curcumin is a promiscuous protein binder.

**Fig. 18:**
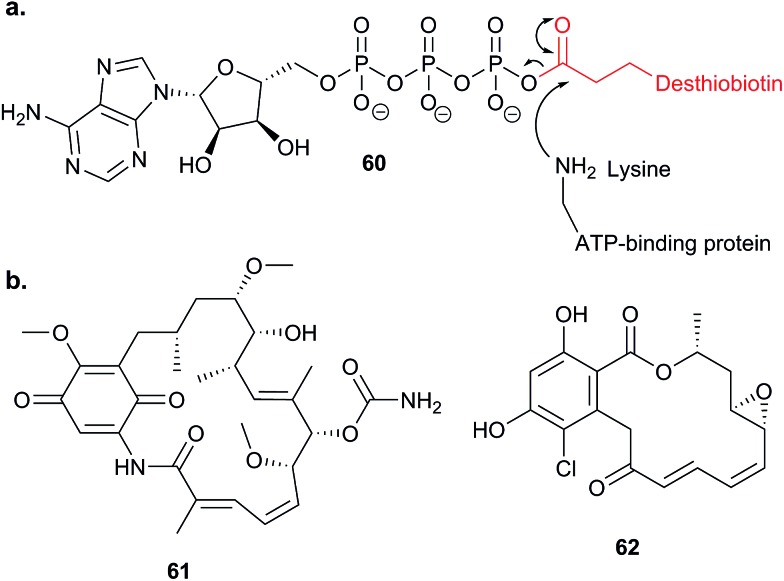
(a) Reaction of lysine in active site of ATP-binding protein with ATP analogue probe **60**.^113^ (b) NPs geldanamycin **61** and radicicol **62**.

Probes that are more specific than general cysteine-reactive compounds for particular subsets of proteins have also been applied in competitive ABPP. Many cellular proteins bind ATP, such as kinases and other ATPases; these include the chaperone HSP90, an important potential drug target in cancer. Nordin *et al.* applied an ATP acyl phosphate probe **60**,^112^ which binds to the ATP pocket and acylates nearby lysine residues with a desthiobiotin tag, *in vitro* (Fig. 18).^113^ Following enrichment, digest and LC-MS/MS, labelled peptides were identified directly based on MS^2^ fragment spectra, aided by an inclusion list to improve detection. Competition with ATP was used to distinguish binding of the probe non-specifically to surface exposed lysines and binding in ATP sites. With this sensitive set-up in place, Nordin *et al.* profiled the ability of two NPs geldanamycin **61** and radicicol **62** (Fig. 18), as well as several synthetic compounds, to inhibit HSP90 paralogs *via* binding to the ATP pocket. The compounds were applied to live cells and the authors also searched for off-targets of the compounds amongst other ATP-binding proteins.

**Fig. 19:**
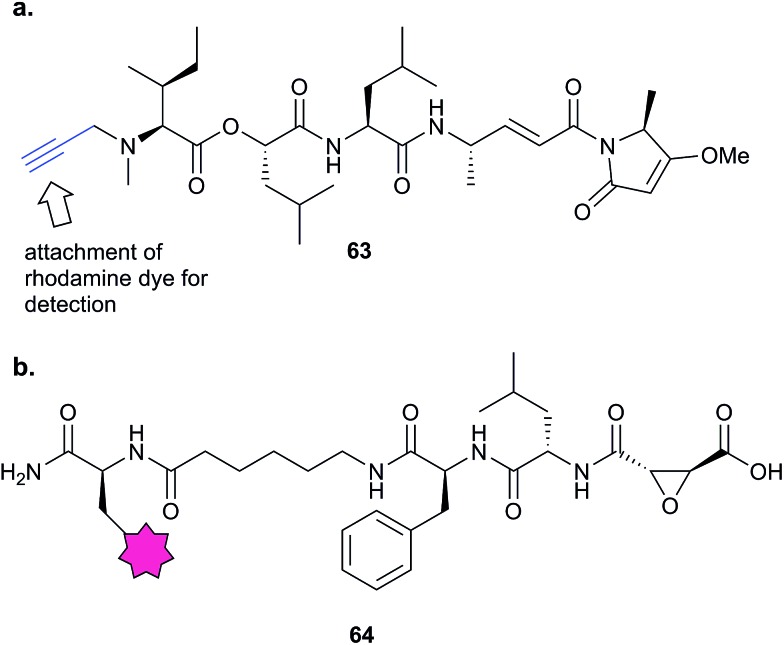
(a) Symplostatin 4 precursor probe **63**. (b) E64-inspired protease probe **64**.^114^

In 2012, Stolze *et al.* combined competitive ABPP and fluorescent probes to reveal the mode of action of symplostatin 4, a secondary metabolite from cyanobacteria with potent anti-malarial activity.^114^ Symplostatin 4 features a potentially protein-reactive Michael acceptor and methyl-methoxypyrrolinone (mmp) unit. SAR studies first demonstrated the importance of the mmp unit for anti-parasitic activity, then the authors further characterised the phenotype arising from treatment of the malaria parasite *Plasmodium falciparum* with symplostatin 4 and derivatives. They observed a defect in the food vacuole, suggestive of a block in haemoglobin degradation. They synthesised probe **63** (Fig. 19) and used the alkyne tag to attach a rhodamine fluorophore. Several proteins were covalently labelled with the rhodamine dye-coupled symplostatin 4 probe, leading the authors to hypothesise that proteases called falcipains, which play roles in parasite development within the red blood cell, were inhibited by the compound. Competitive labelling experiments using probe **64** based on the epoxide-containing cysteine protease inhibitor E-64 showed that symplostatin 4 inhibited specific members of this protease family. Quantification of the labelling revealed the sensitivities of the different proteases to the NP.

**Fig. 20:**
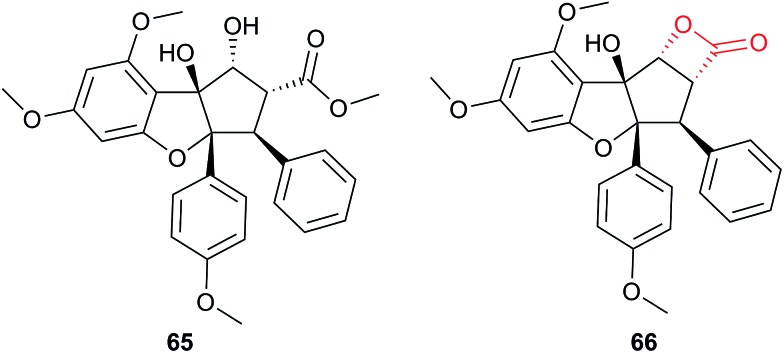
NP rocaglate **65** and β-lactone-functionalised derivative **66**.^16^

Competitive ABPP is intuitively most applicable to reactive NPs. A structural modification that converts an unreactive NP to a reactive one may change the bioactivity or mode of action, and so is a risky strategy for target identification. However, there has always been interest in generating modified versions of NPs and recently Porco, Cravatt and co-workers studied a derivative of the NP rocaglate **65** incorporating a reactive β-lactone moiety (**66**, Fig. 20).^16^ Rocaglates have diverse bioactivity and β-lactones have proven value as electrophilic traps for nucleophilic enzyme residues.^52^ Hypothesising that the β-lactone would direct the compound to enzymes possessing nucleophilic residues, the authors conducted competitive ABPP against a promiscuous fluorophosphonate probe. SILAC quantification of enriched proteins with and without competition revealed four out of 40 serine hydrolases that were specifically and dose-dependently inhibited by the novel compound. Competition also worked well *in situ*, suggesting that the new rocaglate derivative could serve as a tool compound for future studies on specific serine hydrolases.

**Fig. 21:**
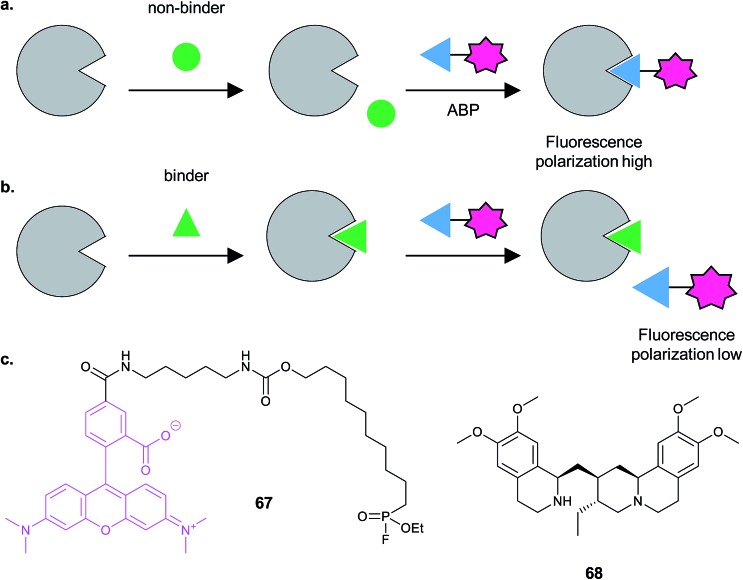
FluoPOL ABPP screening. (a) If a compound does not bind the protein in the assay, the activity-based probe (ABP) can bind, resulting in highly fluorescently polarised light because the fluorophore tumbles slowly. (b) In contrast, if a compound binds, the ABP does not and tumbles rapidly in solution, resulting in low fluorescence polarisation. Figure adapted from Bachovchin *et al.* ^115^ (c) Fluorophosphonate probe **67** and NP hit emetine **68**.^115^

Finally, Cravatt and co-workers developed a high-throughput competitive screening platform termed fluopol-ABPP to screen an enzyme *in vitro* for compounds that bind.^115^ The method works on the premise that if a relatively large ABP functionalised with a fluorophore is attached to a protein of interest, the extent of depolarisation of polarised light emitted by the excited fluorophore will be low. If instead of the probe, a competitor compound is bound, the probe will be free to tumble rapidly in solution and will emit depolarised light (Fig. 21). Bachovchin *et al.* applied this approach to the putative serine hydrolase RBBP9. Approximately 18 000 compounds were screened using RBBP9 and rhodamine-functionalised fluorophosphonate probe **67** that binds serine hydrolases, identifying several hits including the bioactive natural product emetine **68**. Gel-based fluorescence ABPP was further used to refine and study the hits.

## 4 Conclusions

The main strength of chemical proteomics is its ability to provide an unbiased, global and quantitative analysis of protein binding partners. By combining a cell-permeable probe with chemical proteomics, often facilitated by a two-step bioorthogonal ligation protocol, it is possible to work in live cells (‘*in situ*’) and with endogenous protein levels. Probes can also be applied in diverse systems. And using sensitive in-gel fluorescence detection with well-characterised probes, or using quantitative proteomics, it is possible to quantify dose–response, response to inhibitor or competitor and even measure IC_50_ of probe–protein binding directly in the cell.

These advantages make chemical proteomics a very powerful method for mode of action studies. However, as with all technologies, there are limitations and difficulties. We have mentioned many of these in the main part of the review, but in the following section we will explicitly discuss the main problems and some potential solutions.

### 4.1 Limitations, pitfalls and potential solutions

One common concern in chemical proteomics that we have highlighted throughout this review is how to deal with promiscuity and background binders. There are two issues here: firstly, at high concentrations, photoreactive and electrophilic probes will almost always label proteins non-specifically to some extent, and some proteins may be probe-specific hits (*i.e.* not targets of the parent compound). Secondly, some proteins will bind non-specifically to affinity resin, and there may be background from the CuAAC reaction, where used.

The second problem is often addressed by comparison with controls combined with quantitative proteomics. However, it can still be challenging to deal with both highly abundant protein hits (that may well be present in controls too) and low abundance proteins that are masked by background. Furthermore, choosing a cut-off, even with quantitative data, is difficult. Some resources are available to help assess whether a putative hit is a ‘sticky’ or abundant background binder; for example, in one study, the background binders from different resins and affinity enrichment protocols were analysed.^116^ The issue of background is in part due to the indirect nature of most chemical proteomics identification strategies: in general proteins are identified as hits by their enrichment in probe-treated sample over some control and not by direct identification of probe modification. As discussed here, use of a cleavable linker is one approach to this problem; examples discussed above include identification of the sites of modification of cysteine-reactive probes,^40^ the use of isotopically labelled azide reagents to capture peptides from proteins labelled with a reactive lipid probe^92^ and the identification of sites of aspirin alkylation.^79^ Such linkers work best with probes that are incorporated metabolically at defined positions in a protein or probes that react predictably with certain residues (*e.g.* cysteine) and without excessive fragmentation. However, some of these limitations may be overcome as *de novo*-aided sequencing approaches improve and MS instruments become increasingly sensitive.

The issue of probe promiscuity is difficult to deal with because it is so context-dependent. Optimising probe concentration and other labelling conditions can sometimes help, and quantifying enrichment in the presence and absence of a competitor (typically the parent NP) is one approach widely used to test whether a protein is a probe-specific hit. This approach was used to help identify the specific targets of the NPs zerumbone,^91^ eupalmerin acetate,^89^ hypothemicin^80^ and fimbrolides,^85^ for example. Multiple probes with modifications at different positions can be used to reduce the risk of probe-related off-targets and to ensure coverage of as many targets as possible (an approach used to identify possible targets of acivicin, for example).^87^ In PAL, choice of photoreactive group may also have an influence on the profile of hits, although very few comparative studies exist, and it can also be challenging to introduce a photolinker into a small scaffold without affecting compound activity. One recent study analysed the hits of simple compounds incorporating a benzophenone, aryl azide or alkyl diazirine photocrosslinker, as well as alkyne tag, with the aim of providing a list of photolinker-specific targets.^117^ However, this study applied gel-based proteomics, which as discussed above suffers from several technical limitations. Further work in this area may be helpful in providing resources to aid researchers in assessing whether putative targets are genuine or related to the photocrosslinker moiety itself. Promiscuity of photoprobes is a common challenge: as discussed above in the context of kinase inhibitors, it is difficult to identify ‘true’ binders against a background of likely probe-specific targets. In our experience, chemical proteomics experiments, particularly those with technical challenges (*e.g.* photocrosslinkers) or where many proteins are addressed, benefit from the following: (1) the application of quantitative proteomics and rigorous statistical evaluation of data; (2) control experiments, again performed quantitatively *e.g.* competition with parent compound; (3) follow-up validation of putative targets.

Finally, newly described alternative approaches, such as CETSA (cellular thermal shift assay)^118^ or TPP (thermal proteome profiling),^119^ for measuring compound–protein binding in cells complement chemical proteomics and are more readily applicable to compounds with non-covalent binding modes. CETSA or TPP relies on the fact that binding of a compound often stabilises the target protein in a manner that can be detected by a shift in the melting temperature of that protein. Thus, lysates or cells are treated with a compound, and sample aliquots heated to different temperatures; the aliquots are then lysed if necessary and centrifuged to remove aggregated (denatured) proteins; the soluble portion is analysed to detect the amount of undenatured protein remaining. These approaches are only applicable to protein–ligand interactions showing a thermal shift and, currently, to soluble proteins, but are nonetheless very powerful.

Linking target binding with bioactivity, and dealing with pleiotropic effects are broad challenges faced by any approach that seeks to identify the proteins that NPs bind. Structure–activity relationship studies (*e.g.* comparing the effects of compounds with different levels of bioactivity) and in-depth biological characterisation are typically used for this next level of mode-of-action deconvolution.

### 4.2 Current and future challenges

In 2012, Moellering and Cravatt proposed that chemical proteomics can help bridge the gulf between early drug discovery efforts that focus on identifying interesting drug targets, often genetically, and later efforts where the emphasis is on the chemistry or pharmacology of small molecules.^120^ They note that chemical probes provide a way to detect engagement of a compound with its target protein in living systems, and suggest that this strength could be leveraged to bring chemistry-driven approaches into contact with early stages of drug discovery – validating drug targets chemically, identifying novel druggable proteins, and clarifying the mode of action of compounds identified in phenotypic screens. Although chemical proteomics has made huge contributions to our understanding of particular enzyme classes, large portions of the proteome, and many organisms, remain unexplored. It is also interesting that most clinically approved drugs target membrane receptors,^121^ but that relatively few chemical probes have been developed to address this large class of proteins. Affinity chromatography and chemical proteomics in cell lysates are difficult to apply to membrane proteins since cell lysis, particularly if detergents are used, can seriously disrupt these proteins. Other techniques such as CETSA/TPP also currently cannot address membrane proteins. However, *in situ* chemical probes should, in principle, be applicable to capturing membrane targets. Nevertheless, the vast majority of *in situ* chemical probes reported so far target either enzymes with fairly well-defined pockets (proteases, kinases) or are promiscuous electrophiles that bind soluble metabolic enzymes; this is likely due to the field's historical focus on activity-based probes. Several photoaffinity natural product-based probes that target membrane proteins have been reported, including mimics of vancomycin and a peptidomimetic, but the number of examples is still small. The lack of chemical probes targeting membrane receptors may be due to the facts that these proteins typically bind ligands non-covalently (necessitating the use of photoreactive probes or probes designed to react with a non-catalytic nucleophile in the binding pocket), often have multiple active and inactive conformations complicating ligand binding, and are typically of low abundance. As illustrated by the examples given above, with photoaffinity labelling it is particularly challenging to tease out true targets against background labelling, and this may have thus far hindered the development of probes for low abundance membrane receptors. However, *in situ* chemical proteomics is uniquely positioned to tackle this challenge. Improved sensitivity of mass spectrometry instrumentation may aid in the detection of low abundance hits or allow lower concentrations of probe to be used (which can reduce non-specific binding and hence off-targets) and focused efforts to mine chemical space should yield more specific high affinity probes. Design of covalent probes for G-protein coupled receptors is also an active area of research, for example.^122^ Natural products provide a rich resource of potential probes that could be applied in underexplored areas of chemical proteomics.

Chemical proteomics, by its nature, focuses on proteins, but small molecules also act on other biomolecules (DNA, RNA) or structures (membranes). However, many of the same principles of chemical proteomics could be developed for direct detection of binding of small molecules to oligonucleotides. For example, clickable Pt(ii)-complexes were used to identify rRNA interactors of the drug picoplatin in *Saccharomyces cerevisiae*.^123^ Studies applying such complexes were recently reviewed by White *et al.* ^124^

Finally, the indirect nature of many identification strategies is a limitation that we have discussed in depth here. However, cleavable linkers and advances in quantitative proteomic technology continue to improve. We anticipate that efforts to make these technologies more broadly applicable and the development of innovative tools will continue to be at the cutting edge of chemical proteomics.
